# Cell-cycle inhibition preserves robust development but rebalances lineages in mouse gastruloids

**DOI:** 10.1016/j.isci.2026.118084

**Published:** 2026-11-02

**Authors:** Maxine Leonardi, Yves Paychère, Felix Naef

**Affiliations:** 1 Laboratory of Computational and Systems Biology, Institute of Bioengineering, Faculty of Life Sciences, École Polytechnique Fédérale de Lausanne (EPFL), Lausanne, Switzerland

**Keywords:** cell cycle, gastruloid, gastrulation, development, cell differentiation, cell proliferation, stem cells, scRNA-seq, modeling

## Abstract

Cell differentiation and proliferation are fundamental to multicellular development. While studies in various non-mammalian species show that development can proceed despite disrupted cell-cycle progression, the extent to which normal cell-cycle dynamics are required in mammals remains unclear. Using mouse gastruloids, we examined the effects of cell-cycle inhibition on development. Despite near-complete growth arrest, gastruloids still underwent symmetry breaking, elongation, and germ-layer specification, indicating that core differentiation programs are robust to growth inhibition. However, microscopy and single-cell transcriptomics revealed consistent alterations in cell-type proportions, including delayed differentiation and reduced mesodermal populations. To investigate these changes, we used the differential kinetics of unspliced and spliced cycling transcripts to compare proliferation rates between cell types. While differences in proliferation partly explained the imbalance, our analysis showed that cell-cycle perturbations also modulate lineage-specific differentiation timing and efficiency, highlighting a regulatory role of cell-cycle dynamics in mammalian development.

## Highlights

• Gastruloid development remains robust despite palbociclib-induced growth arrest
• Growth arrest rebalances lineages, reducing mesoderm and delaying differentiation
• Cell type proliferation rates alone do not explain the observed lineage rebalance

## Graphical abstract

**Figure:**
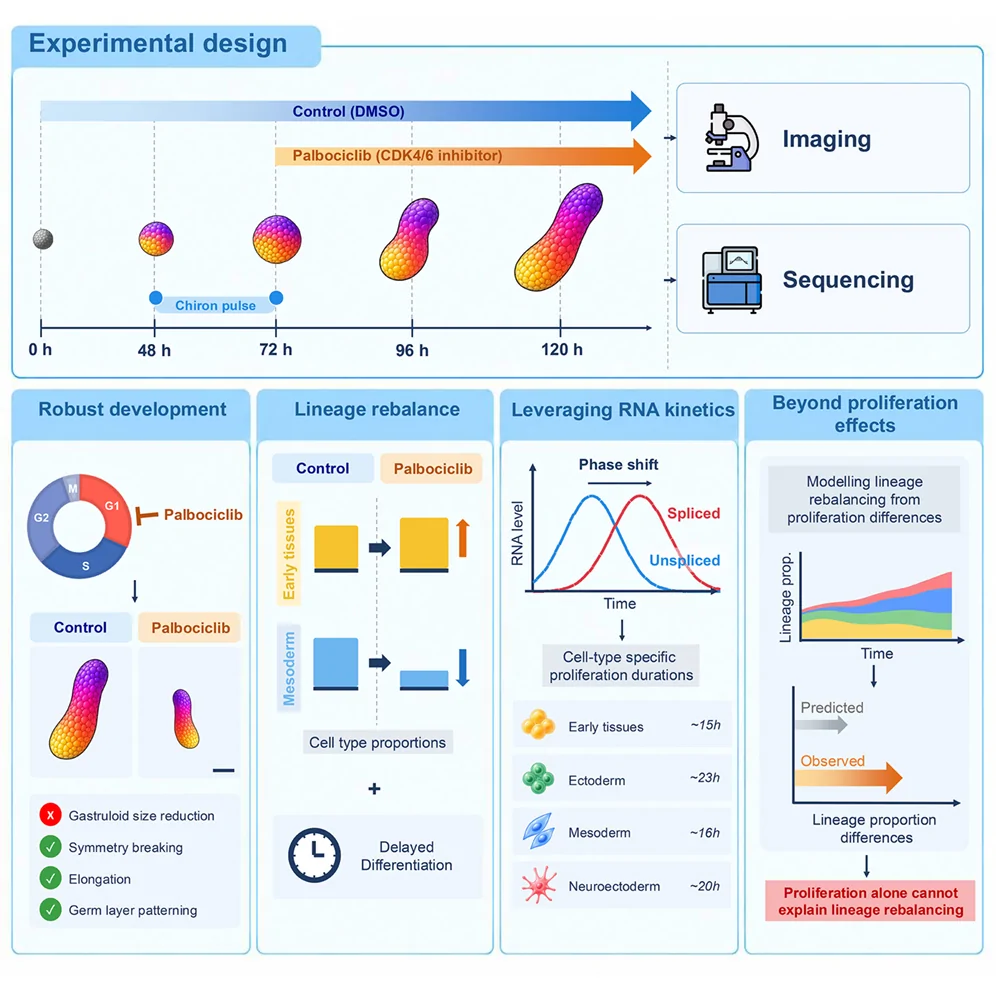

## Teaser

Evolutionary biology; Cell biology; Developmental biology

## Introduction

The cell cycle is a fundamental process that governs the growth, development, homeostasis, and regeneration of all living organisms, and its dysregulation is a hallmark of many diseases.^1^^,^^2^ Beyond the primary role of the cell cycle in growth control, its direct involvement in regulating pluripotency and cell fate commitment has become increasingly clear. One of the most consistent observations is that higher potential for pluripotency coincides with a faster cell cycle, often marked by a shortened G1 phase.^3^ At the molecular level, core pluripotency factors directly promote the cell cycle by acting upstream of key cell-cycle regulators.^4^^,^^5^^,^^6^^,^^7^^,^^8^^,^^9^^,^^10^^,^^11^^,^^12^ Fast cell cycles are not only a hallmark of pluripotent cells but also actively sustain this state. Indeed, cell-cycle regulators are, in turn, direct regulators of pluripotency factors.^13^^,^^14^^,^^15^^,^^16^^,^^17^ Therefore, inhibition or knockout of many CDKs or cyclins leads to spontaneous differentiation,^18^^,^^19^^,^^20^^,^^21^^,^^22^^,^^23^ while their overexpression can delay lineage commitment.^22^^,^^24^

Cell-cycle control remains essential throughout the transition from pluripotency to differentiation. As cells differentiate, the cell-cycle duration extends with the G1 phase becoming longer, providing a window of opportunity to integrate external differentiation signals, partly through chromatin restructuring.^25^^,^^26^ This extended G1 phase along with the proteins that regulate it have been shown to play a crucial role in the commitment toward multiple lineages, such as mesoderm,^27^^,^^28^ neural,^29^^,^^30^^,^^31^^,^^32^ and endothelial tissues.^33^ Moreover, the timing of differentiation cues within G1 phase also influences cell fate: exposure to such cues in early G1 promotes mesendodermal fate, whereas cues in late G1 favor neuroectoderm, partly via CDK-dependent regulation of cell signaling pathways.^34^^,^^35^ Beyond the cell-cycle regulation of individual cell fates, early development in many species, such as zebrafish, *C. elegans*, and *Xenopus*, is characterized by synchronized and spatially coordinated cycle events.^36^^,^^37^^,^^38^ Similarly, a functional role of the spatial organization of cell-cycle phases has been identified in mouse development, particularly during somitogenesis.^39^^,^^40^

These works highlight a clear interplay between the cell cycle and cell-fate decisions. Maintaining the balance between pluripotency and differentiation is essential during early embryogenesis. Therefore, altering cell-cycle progression should potentially derail this balance and impair normal development. However, evidence across model organisms suggests that early development is surprisingly robust to cell-cycle perturbations.^41^ Indeed, in several systems, early development proceeds despite arrested proliferation: in *C. elegans*, cell cycle mutants that abolish mitosis can still initiate transcriptional programs of differentiation on time.^42^ Similarly, in *Xenopus*, blocking DNA replication does not prevent morphogenetic events like neurulation.^43^^,^^44^^,^^45^ In zebrafish, embryos can still undergo axis elongation and form blood, muscle, and a beating heart even when cell division is blocked from gastrulation onward. However, this arrest in proliferation leads to defects in morphogenesis, cell migration, and tissue organization.^46^^,^^47^ A recent single-cell RNA sequencing (scRNA-seq) study has further confirmed that differentiation of all major embryonic tissues can occur in the absence of cell division.^48^ However, the proportions of the resulting cell types were altered significantly, with differences of up to 10-fold, and some lineages showed a delay in differentiation, indicating that while proliferation is not strictly required for lineage specification, it does influence the pace and balance of developmental processes.

In mouse, most cyclins and CDKs are dispensable for early development, with knockouts causing only limited or tissue-specific defects.^49^^,^^50^^,^^51^ However, these mild phenotypes likely arise from functional redundancy among cell-cycle regulators, which maintains near-normal cell cycle dynamics during embryogenesis, rather than from an inherent robustness of development to cell cycle perturbations. Effective perturbations of the cell cycle have also been investigated in mouse embryos through transient chemical arrest of proliferation, which significantly reduced cell numbers but still allowed embryos to undergo morphogenesis, although the long-term consequences include impaired growth and fertility.^52^ In other studies, altering cell cycle dynamics in the mouse embryo has been shown to cause defects in specific tissues, notably in the neuronal lineage,^53^^,^^54^ or in later developmental processes such as somitogenesis, where cell-cycle arrest leads to abnormalities in somite length.^40^ In humans, mutations in CDKs or their inhibitors have been linked to developmental disorders such as microcephaly or gigantism.^55^^,^^56^^,^^57^ Moreover, *in vitro* studies using human embryoid bodies indicate that altering cell cycle regulators can shift lineage allocation.^58^ Together, these findings illustrate a complex, context-dependent relationship between proliferation and mammalian development and highlight that the overall influence of the cell cycle on cell-fate specification remains poorly understood, particularly in mammalian development.

Here, we use mouse gastruloids, an *in vitro* model of gastrulation,^59^^,^^60^^,^^61^^,^^62^^,^^63^^,^^64^^,^^65^ to investigate the consequences of cell cycle inhibition on mammalian development. Based on the established links between cell-cycle regulation and cell-fate decisions, we hypothesized that inhibiting cell-cycle progression during gastruloid development could potentially perturb morphogenetic processes and alter the differentiation and commitment of cells toward the emerging developmental lineages. Combining microscopy and scRNA-seq, we show that despite near-complete proliferation arrest after drug treatment, gastruloids preserved key developmental features such as symmetry breaking and elongation, and generated cell types from all three germ layers. However, cell-type proportions were quantitatively altered in growth-arrested gastruloids consistently across all data modalities, with delayed differentiation in specific lineages and a rebalancing of the distributions of cell differentiation trajectories. To investigate the underlying cause of these differences, we compare unspliced and spliced RNA profiles to map cell cycle rates along the different differentiation lineages. This demonstrated that differences in proliferation rates partially accounted for the changes observed in growth-arrested gastruloids, but were insufficient to fully explain them, arguing that the cell cycle also likely modulates the efficiency of differentiation through another mechanism. Together, these findings suggest that although the cell cycle is not required for the developmental processes at play during gastrulation, it likely contributes to lineage balance and timing within mouse gastruloids.

## Results

### Cell-cycle-inhibited gastruloids break symmetry, elongate, and recapitulate patterned marker-gene expression but show altered cell-type proportions

To investigate how modulating cell cycle kinetics influences early mammalian development, we generated gastruloids from mouse embryonic stem (ES) cells using standard protocols^59^ and treated with pharmacological inhibitors targeting key regulators of proliferation, namely CDK4/6, CDK2, CDK1, and mTOR from either 48 h or 72 h after aggregation (A.A.) onward (Figure 1A). These conditions and doses were titrated to slow down the cell cycle without inducing gastruloid death. In addition, a Wnt pathway inhibitor was used to probe abnormal development, as it is expected to impair gastruloid development by acting directly upstream of T, a major regulator of mesendodermal fated cells. Gastruloid growth and morphology were monitored by brightfield imaging every 24 h (Figure 1B; Figure S1A), and samples were collected at 120 h A.A. for subsequent immunofluorescence staining (IF) (Figure 1C).

**Figure 1:**
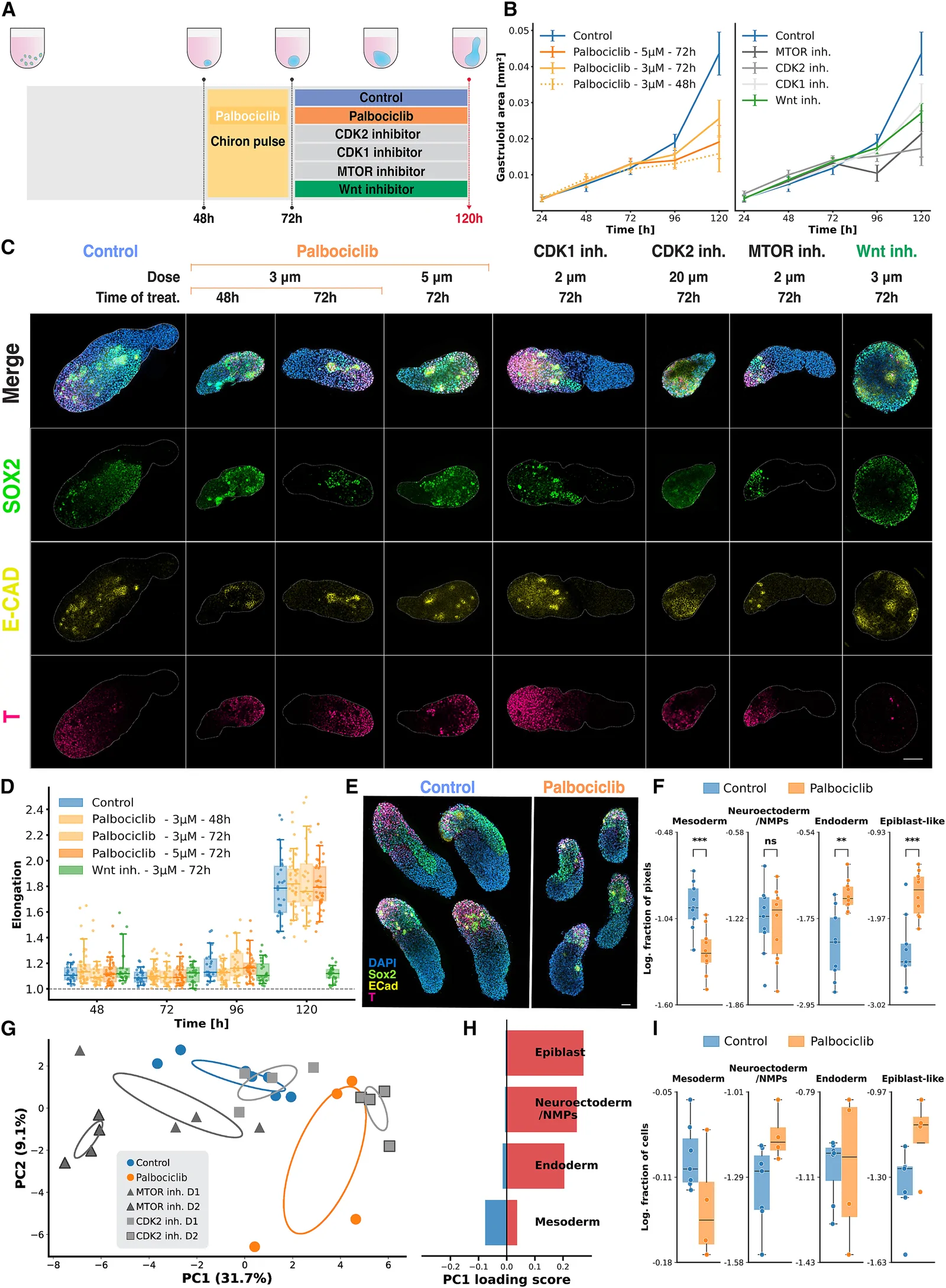
Cell-cycle inhibited gastruloids break symmetry, elongate, and recapitulate patterned marker gene expression, but show altered cell-type proportions (A) Experimental design: gastruloids were generated by aggregating 300 mouse ES cells and treating them with Chiron from 48 to 72 h. From 48 or 72 h onward, they were treated with either DMSO (control), CDK4/6 inhibitor (palbociclib), CDK2 inhibitor (NU6102), CDK1 inhibitor (RO3306), mTOR inhibitor (Torin1) or Wnt inhibitor (porcupine). Gastruloids were imaged with the Operetta microscope every 24 h and collected at 120 h for subsequent immunofluorescence stainings. (B) Operetta-based quantification of gastruloid area progression reveals substantially reduced growth under CDK4/6 (left) and other cell cycle and Wnt inhibitor treatments (right). *n* ≥ 20 for all conditions. Error bars represent the standard deviation (SD). (C) IF of gastruloids in control and drugged conditions. Stainings for T, SOX2, and E-CAD show that cell-cycle inhibition does not prevent cell-type marker expression and patterning, despite strong size reduction. Scale bars, 100 μm. (D) Quantification of elongation reveals strong impairment under Wnt inhibition, but no effect with CDK4/6 inhibition. *n*>=20 for all conditions. Boxplot shows median, 25th–75th percentiles (box), and whiskers extending to the 5th/95th percentiles; all data points are shown (jittered). (E) Further representative staining of T, SOX2, and E-CAD in control and palbociclib-treated gastruloids (5 μM, from 72 h), the conditions selected for subsequent quantification. Scale bars, 50 μm. (F) Comparison of cell type composition from IF stainings in control and palbociclib-treated gastruloids. Quantification shows a significant reduction of mesodermal cells and an increase of early tissue populations retaining epiblast-like pluripotency. *n*_*control*_ = 9; *n*_*palbociclib*_ = 10. Statistical significance was assessed using a two-sided Welch’s *t* test on log-transformed data, with ∗*p* < 0.05, ∗∗*p* < 0.01, ∗∗∗*p* < 0.001,∗∗∗∗*p* < 0.0001 indicating significant differences. Boxplot shows median, 25th–75th percentiles (box), and 1.5× IQR (interquartile range) whiskers. (G) PCA of cell type marker genes from bulk sequencing of single gastruloids across control and inhibitor treatments. Samples cluster by condition along PC1, reflecting distinct cell type marker expression profiles. *n*_*control*_ = 7; *n*_*others*_ = 4. (H) PC1 gene loading scores of cell type marker genes. The score (sum of squared loading) reflects the contribution of marker genes from each cell type to PC1. Epiblast, endodermal, and neuroectodermal markers load positively on PC1. Mesodermal markers load negatively on PC1. (I) Cell type proportions from bulk RNA sequencing after cell type deconvolution with Scaden^66^ reveals trends consistent with immunostaining quantifications (E). Boxplot shows median, 25th–75th percentiles (box), and 1.5× IQR whiskers.

Across all cell-cycle inhibition conditions, gastruloid growth was strongly reduced after treatment compared to controls, with consistent results across replicates (Figure 1B; Figure S1B). In particular, the higher doses of the CDK4/6 inhibitor palbociclib (5 μM) produced gastruloids approximately 3 times smaller in area. In this condition, the gastruloid area continued to increase slightly over time, even under drug treatment. However, this likely reflects their elongated shape at 120 h, which results in a larger projected area for the same volume rather than an increase in cell number. Consistent with this interpretation, quantification of cell counts over time showed no net growth after treatment, indicating a balance between proliferation and apoptosis in the presence of the drug (Figure S2E).

Interestingly, these growth-arrested gastruloids still underwent symmetry breaking despite their reduced size. This process, defined as the emergence of a molecular or morphological asymmetry from an initially homogeneous cell population, was evident from the formation of an axis and the polarized expression of marker genes in all cell-cycle inhibited gastruloids. Elongation, quantified as the ratio between the gastruloid’s major and minor axes (Figure S1C), showed no significant difference between control and palbociclib-treated gastruloids, whereas Wnt inhibition completely abolished elongation, with gastruloids that stayed spherical until 120 h (Figure 1D). A similar reduced-growth but preserved-elongation phenotype was observed in gastruloids generated from E14 mouse ES cells, indicating that this response is not specific to the CGR8 cell line (Figures S1D–S1F). Consistently, treatment with the structurally distinct CDK4/6 inhibitors ribociclib and abemaciclib similarly reduced gastruloid growth by approximately 2.5-fold without significantly affecting elongation, supporting that this phenotype is not specific to palbociclib (Figures S1G and S1H). These results indicate that normal cell cycle dynamics are not required for symmetry breaking or axis formation in gastruloids.

Moreover, cell type markers of all the germ layers (combination of T, SOX2, and E-CAD) were still expressed in all cell cycle inhibited gastruloids at 120 h, forming clusters and patterns similar to those observed in control gastruloids (Figure 1C). In contrast, Wnt-inhibited gastruloids almost completely failed to express the mesodermal marker T, nor did they show regions of E-CAD expression without SOX2, an endodermal marker, indicating the expected disrupted mesendoderm formation. Together, these findings suggest that cell-cycle inhibition does not markedly impair either the emergence or the patterning of distinct germ layer cell types in gastruloids.

In the following analyses, one representative cell-cycle inhibition condition (CDK4/6 inhibition at 5 μM from 72 h) was selected for further quantitative assessment of cell-type proportions. Among the tested perturbations, CDK4/6 inhibition was selected for this in-depth analysis because the G1 phase, and particularly the G1/S transition, is the cell-cycle window most consistently linked to pluripotency exit, differentiation competence, and lineage choice.^25^^,^^26^^,^^27^^,^^29^^,^^34^^,^^35^ The 72 h time point was chosen because it coincides with the critical window for symmetry-breaking commitment identified in dissociation-reaggregation experiments,^67^ making it a biologically relevant stage at which to assess the consequences of cell-cycle inhibition on subsequent differentiation.

In this condition, the effects of the inhibitor on cell-cycle dynamics were characterized using several complementary approaches. We first characterized the effect of palbociclib in undifferentiated CGR8 ES cells cultured in 2D 2i/LIF conditions,^68^ where it reduced proliferation in a dose-dependent manner and led to near-complete growth arrest at 5 μM (Figure S2A). Consistent with the marked reduction in proliferation, 5 μM palbociclib also reduced the proportion of pRB-positive ES cells by approximately 3-fold (Figures S2B and S2C), confirming that this concentration effectively inhibits CDK4/6 activity. Having validated the drug’s activity in 2D, we then characterized its effects on cell cycle dynamics within gastruloids themselves. This included Fucci(CA) imaging and flow cytometry analyses performed throughout gastruloid development (Figures S2D–S2H). In this reporter, CDT1 expression marks cells in the G1 phase, GEMININ expression marks cells in the S phase, and co-expression of both indicates that cells are in the G2/M phase. Quantification from microscopy images and flow cytometry data both showed a time-dependent accumulation of G1-phase cells in palbociclib-treated gastruloids compared to controls (Figures S2F and S2H). This phenotype was further confirmed by DNA content profiles derived from DAPI fluorescence in the flow cytometry experiments (Figures S2G and S2K). These findings are consistent with CDK4/6 acting as a G1/S checkpoint regulator. Although an enrichment of G1 cells is observed, other cell-cycle phases are still represented in the drugged gastruloids, indicating that cell cycle is slowed down rather than completely blocked in the G1 phase.

To assess whether other phases of the cell cycle were also affected by cell cycle inhibition, proliferation assays were performed by pulsing 120 h gastruloids with 5-ethynyl-2′-deoxyuridine (EdU) for 2 h. Imaging and flow cytometry analyses both revealed a strong decrease in EdU incorporation in the inhibited condition (Figures S2I and S2K–S2M). Moreover, the EdU-positive cells that remained exhibited lower fluorescence intensity than controls (Figure S2M), suggesting that not only fewer cells entered S phase but also that those cells replicated their genomes more slowly. Together, these results demonstrate that CDK4/6 inhibition induces a globally slower cell-cycle progression, with a particular cell accumulation in the G1 phase. Moreover programmed cell death, which has been showed to be a main driver of cell competition in gastruloid,^69^ was variable under the CDK4/6 inhibition between gastruloids, but tended to be slightly higher overall than in controls, as shown by activated caspase staining (Figures S2J and S2N).

Having established the effects of CDK4/6 inhibition on cell-cycle progression, we next investigated whether this perturbation also impacted the cell-type composition of gastruloids using cell marker IF staining (Figure 1E). Automated quantification of immunostainings using a pixel-based classification (Figures S1I and S1J) suggested significant shifts in lineage proportions. Specifically, CDK4/6 inhibition reduced the fraction of T-positive mesodermal cells while increasing both E-CAD-positive populations and SOX2 and E-CAD-positive populations characteristic of endoderm and epiblast-like early tissues, respectively (Figure 1F).

To complement these observations, we next asked whether these lineage shifts were also reflected at the transcriptional level. Therefore, we performed bulk RNA sequencing of individual gastruloids at 120 h, in control conditions and in conditions where the cell cycle is inhibited with multiple drugs (CDK4/6 inhibitor, CDK2 inhibitor, and mTOR inhibitor). In the palbociclib-treated condition, differential expression analysis confirmed transcriptional changes related to cell cycle regulation, including the upregulation of D-type cyclins and downregulation of proliferation markers such as *Mki67* and *Pcna* transcripts (Figures S2O and S2P; Table S1). The increased expression of D-cyclins is consistent with the CDK4/6 inhibitor (palbociclib) signature observed previously,^70^ and likely reflects a compensatory response following inhibition of their regulatory partners. Principal component analysis (PCA) of cell-type marker genes separated samples by condition, indicating that marker-gene expression profiles differ between treatments (Figure 1G). Interestingly, CDK4/6 and CDK2 inhibition, both acting on the G1/S checkpoint, exhibited opposite trends to mTOR inhibition relative to the control, suggesting that these pathways may have opposing effects on cell type composition. The gene loadings of principal component one (PC1) indicated that mesodermal markers contributed negatively to this axis, whereas epiblast, neuroectodermal/NMPs (neuromesodermal progenitors) and endodermal markers loaded it positively (Figure 1H). Thus, the positive shift of palbociclib-treated gastruloids relative to controls is consistent with a decrease in mesodermal and an increase in endodermal and epiblast-like transcriptional signatures. Moreover, cell type deconvolution using Scaden^66^ corroborated this, confirming a decrease in mesodermal fractions and enrichment of epiblast-like populations consistent with IF quantifications (Figure 1I).

Taken together, these results show that gastruloid development remains remarkably robust under conditions of cell cycle slow-down. Even when the global gastruloid growth is halted, gastruloids can break symmetry, elongate, and establish patterned germ layer domains. Moreover, expression of the main germ layer markers still emerges, indicating that CDK4/6 inhibition does not completely disrupt differentiation. However, lineage proportions appear altered consistently at both the protein (IF) and mRNA levels, suggesting that while normal cell cycle progression is not essential for the initiation of germ layer formation, it contributes to maintaining balanced lineage specification and may influence the efficiency of fate transitions within developing gastruloids.

### scRNA-seq reveals that cell-cycle inhibition preserves core developmental trajectories but alters lineage balance and delays differentiation in gastruloids

To further examine the consequences of cell cycle inhibition on gastruloid development at the single-cell level and to investigate how the different lineage trajectories unfold over time under growth arrest, we performed scRNA-seq on pooled gastruloids under three previously described conditions (STAR Methods): untreated controls (DMSO), CDK4/6-inhibition (5 μM), and Wnt-inhibition. Samples were collected at 0 h (mESCs) and then every 12 h from 48 to 120 h, with treatments applied from 72 h onward (Figure 2A). This time-resolved design enabled us to capture transcriptional changes across thousands of individual cells and reconstruct gastruloid development from early to late stages in both normal and perturbed conditions.

**Figure 2:**
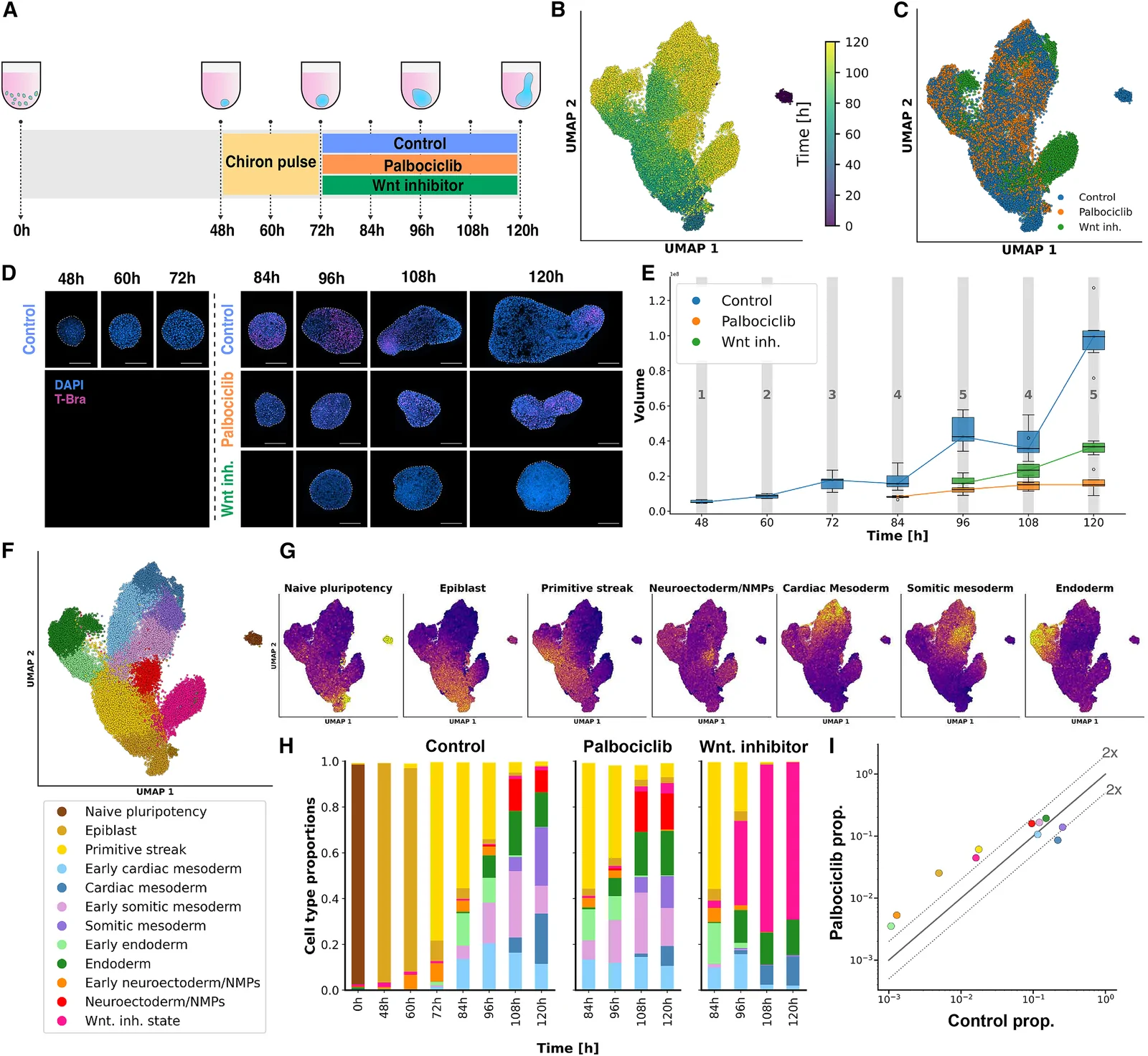
scRNA-seq confirms preserved developmental trajectories but rebalanced cell type proportions in cell cycle-inhibited gastruloids (A) Experimental design: gastruloids were generated by aggregating 300 mouse ES cells and treating them with Chiron from 48 to 72 h. From 72 h onward, they were treated with either DMSO, CDK4/6 inhibitor (palbociclib), or Wnt inhibitor (porcupine). For scRNA-seq, gastruloids were pooled and collected at time points indicated by an arrow. (B) UMAP projection of scRNA-seq data showing cell state evolution over time. (C) UMAP projection of scRNA-seq data shows cell-state distribution over the different conditions. (D) Microscopy of gastruloids from scRNA-seq batches confirms previously observed phenotypes. (E) Gastruloid volume quantification shows growth arrest under CDK4/6 inhibition, leading to a 9-fold smaller size than controls after two days of treatment. (F) UMAP projection of cell-type classification of scRNA-seq dataset shows effective differentiation into neuroectoderm/NMPs, mesoderm, and endoderm trajectories in gastruloids. (G) UMAP projection highlights specific marker gene expression across different cell types. (H) Proportions of the different cell types, stratified by time of collection and conditions reveals similar cell type distribution in control and CDK4/6 inhibitor conditions, but very distinct populations in Wnt inhibitor condition. (I) Comparison of cell-type proportions in control versus CDK4/6 inhibitor conditions, shown in log scale, reveals a rebalancing of cell populations. Dotted lines indicate a 2-fold difference. All boxplots show median, 25th/75th percentiles (box), and 1.5× IQR whiskers.

Imaging of gastruloids from the same batches used for scRNA-seq confirmed the above phenotypes: after treatment onset, palbociclib-treated gastruloids showed no net volume growth, resulting in a final volume approximately 9-fold smaller than controls, yet they still elongated and polarised normally. Conversely, Wnt-inhibited gastruloids failed to elongate and remained spherical (Figures 2D and 2E; Figure S3A). Moreover, palbociclib-treated cells displayed the expected signatures of cell cycle impairment, including D-type cyclin overexpression and enrichment of genes associated with cell cycle-related Gene Ontology (GO) terms (Figures S3C and S3D; Table S2).

Dimensionality reduction of the scRNA-seq dataset revealed a continuous distribution of cell states consistent with progressive differentiation. All samples, except the 0 h time point which formed a distinct cluster corresponding to pluripotent cells, were arranged along a continuum from early to late time points (Figure 2B). When comparing conditions, control and palbociclib-treated cells showed largely overlapping distributions over the uniform manifold approximation and projection (UMAP) space, indicating similar transcriptional trajectories despite growth arrest (Figure 2C). In contrast, Wnt-inhibited cells diverged strongly from controls and formed distinct clusters absent in the control condition.

Annotation of cell types using established lineage marker genes revealed three distinct trajectories that cells can take, corresponding to neuroectoderm/NMPs, mesoderm, and endoderm lineages (Figures 2F and 2G). This organization reflects the emergence of the three primary germ layers during gastruloid development. Analysis of cell-type proportions across conditions revealed a strong effect of Wnt inhibition, with 90% of cells accumulating within a single transcriptional cluster enriched for neuronal signatures (Figure S3B; Table S3). This is consistent with the expected failure to establish a primitive streak-like population due to repression of T expression. In contrast, all germ layer derivatives observed in controls were also present in palbociclib-treated gastruloids (Figure 2H), indicating that cell-cycle inhibition does not prevent lineage specification. While overall cell-type proportions in palbociclib-treated gastruloids remained correlated with controls, measurable shifts in proportion were still present, exceeding 2-fold for some lineages (Figure 2I). These changes included a depletion of mesodermal cells and an increased representation of early progenitor-like states and endodermal cells and ectodermal cells in CDK4/6 condition, confirming prior observations from microscopy and bulk data. The changes in cell-type composition observed at 120 h were already detectable at 108 h in an independent experiment, with persistence of undifferentiated populations and reduced mesodermal cell types becoming more pronounced with longer treatment duration (Figure S3E). Bootstrap resampling of the scRNA-seq data further showed that these differences were robust to cell-sampling variability (Figure S3F; Table S4). Collectively, these findings demonstrate that CDK4/6 inhibition preserves the core differentiation program but likely perturbs lineage balance, resulting in reproducible changes in germ layer composition.

CDK4/6 inhibition appears to affect both cell cycle progression and differentiation processes. However, dimensionality reduction methods like UMAP integrate variation from all sources of cell state and cannot distinguish between the underlying biological processes driving transcriptional changes. Therefore, we sought to disentangle these overlapping sources of variability to better interpret the effects of cell-cycle inhibition. To achieve this, we applied a variational autoencoder (VAE) that separates variance components associated with cell cycle (*θ*-space) from those reflecting other cell states, including differentiation (*z*-space) (Figure 3A).^71^ The *θ*-space representation revealed the characteristic circular manifold of the cell cycle, with RNA velocity fields following this cyclic trajectory (STAR Methods). This representation further enables inference of a cell cycle phase for each cell. When applied to our dataset, the inferred phase distribution correctly identified the excess of G1-phase cells in the cell-cycle inhibited condition (Figure S3G). In contrast, the *z*-space captured the differentiation-related structure of the data, revealing progression of cells from a homogeneous population to distinct branches corresponding to the main germ layers (Figure 3B). A closer examination of cell-density dynamics across the *z*-space provides further insight into how perturbations affect developmental trajectories (Figure 3C). Consistent with previous observations, cells in the control and CDK4/6 inhibition conditions exhibited globally similar temporal progression, whereas Wnt inhibition strongly biased cell-fate decisions toward one cell fate. To compare the propensity of cells to follow specific differentiation trajectories, we applied optimal transport (OT) analysis (STAR Methods), which confirmed that transitions toward mesodermal fates were less frequent under CDK4/6 inhibition (Figure 3D), consistent with the reduced mesodermal representation observed by immunostaining and cell-type quantification (Figure 1F; Figure 2H).

**Figure 3:**
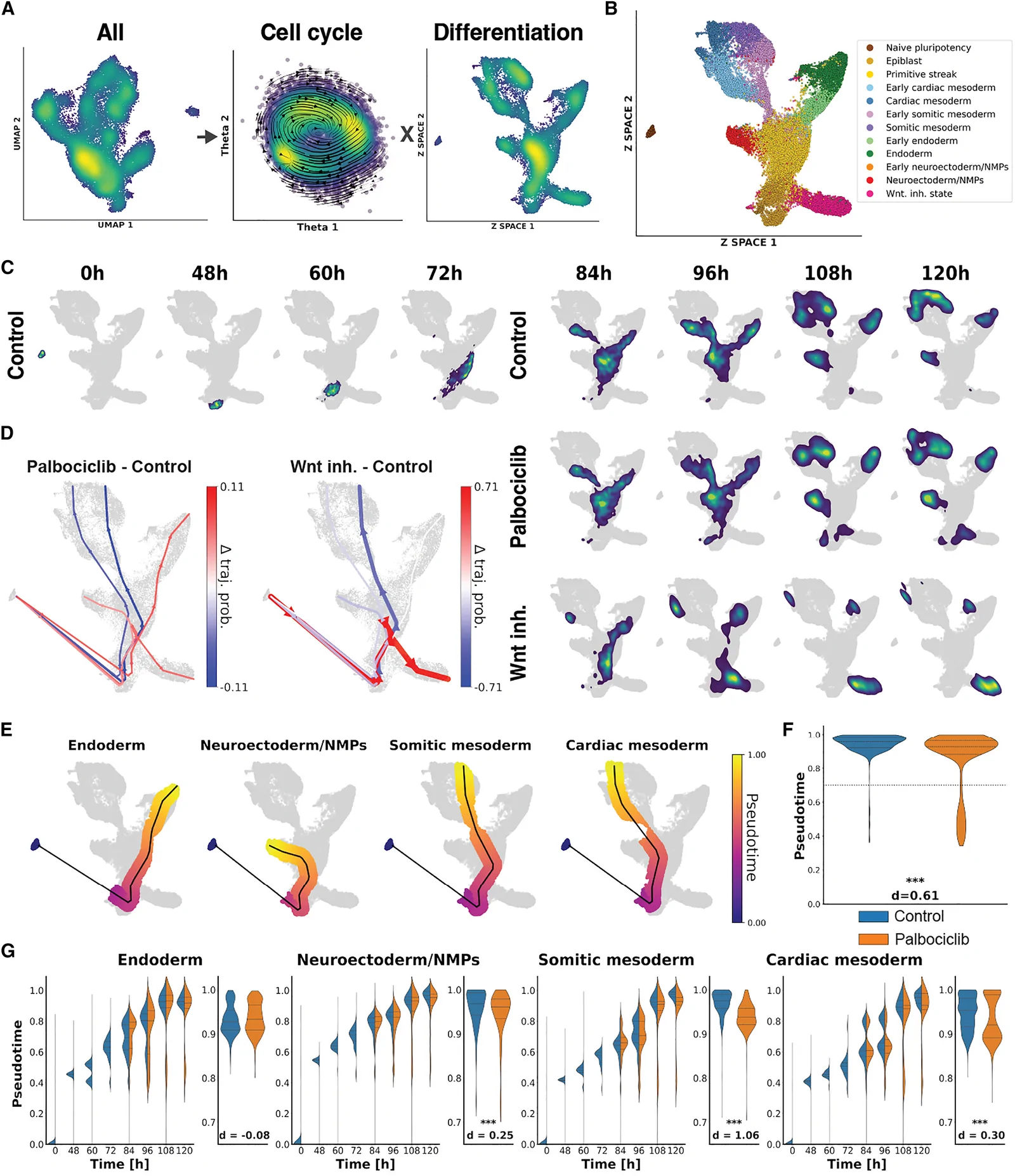
Cell-cycle inhibition in gastruloids biases lineage decisions and delays differentiation (A) Disentangled cell cycle (*θ*-space) and cell differentiation (*z*-space) states in the scRNA-seq data, using a variational autoencoder. (B) UMAP projection of the *z*-space shows the differentiation of cells in endoderm, mesoderm, and neuroectoderm/NMPs branches. (C) Cell density, stratified by time and condition, reveals dynamic changes in cell states during gastruloid development. (D) Comparison of trajectory propensities using optimal transport reveals a biased cell-fate decision in CDK4/6 inhibitor-treated lineages, characterized by reduced mesoderm differentiation. (E) Trajectories and their associated pseudotime describe progression along different developmental paths. (F) Distribution of pseudotime across all lineage trajectories for control and CDK4/6 inh. conditions at 120 h highlights the presence of a less differentiated cell cluster (pseudotime <0.7) in the CDK4/6 inh. treated group. Significance levels are indicated as follows: ∗*p* < 0.05, ∗∗*p* < 0.01, ∗∗∗*p* < 0.001, ∗∗∗∗*p* < 0.0001. “d” denotes Cohen’s effect size. (G) Distribution of pseudotime across different cell type trajectories over time (left) and the associated pseudotime distribution for 120 h gastruloids with pseudotime >0.7 (right) reveal a delay in differentiation, particularly strong in the mesoderm lineages. Significance levels are indicated as follows: ∗*p* < 0.05, ∗∗*p* < 0.01, ∗∗∗*p* < 0.001, ∗∗∗∗*p* < 0.0001. “d” denotes Cohen’s effect size. All violins show density with quartile lines (25th/median/75th); significance (pseudotime >0.7, 120 h) from a two-sided Welch’s *t* test.

To further assess differences in the temporal dynamics of differentiation between the control and CDK4/6 inhibition conditions, pseudotime inference was performed across all trajectories, with higher pseudotime values indicating a more differentiated state (Figure 3E). Comparing pseudotime distributions between conditions, we observed that in palbociclib-treated gastruloids, a distinct population of cells with very low pseudotime values emerged, corresponding to an epiblast-like state absent in controls (Figure 3F). Consistent with this, immunostaining for the pluripotency marker OCT4 at 120 h revealed a significant increase in OCT4-positive cells in palbociclib-treated gastruloids, whereas OCT4 signal was close to background levels in controls (Figures S3H and S3I). This suggests that some cells failed to commit to a lineage or perhaps reverted to a pluripotent state after cell-cycle inhibition.

A second effect we observed was that, even within differentiating populations, the pseudotime distributions of palbociclib-treated cells were globally shifted toward lower values compared with controls, indicating delayed differentiation across lineages. This effect was most pronounced in mesodermal cells but was also evident in neuroectodermal trajectories (Figure 3G). However, endodermal differentiation was not significantly affected by this delay.

Overall, scRNA-seq confirmed that gastruloids exposed to CDK4/6 inhibition retain the ability to generate all three germ-layer lineages, consistent with immunostaining and bulk transcriptomic data. However, lineage balance is altered, with mesodermal cells consistently underrepresented. Moreover, the presence of a persistent undifferentiated population and reduced pseudotime progression indicate delayed or incomplete differentiation under cell-cycle inhibition. Together, these findings show that although normal cell-cycle progression is not required to initiate germ-layer formation, it influences lineage balance and the efficiency of developmental progression in gastruloids.

### RNA velocity and population modeling indicate that cell cycle rate differences alone do not account for CDK4/6-induced cell type rebalancing

Cell cycle inhibition resulted in a redistribution of cell-type proportions in gastruloids, suggesting an interplay between proliferation and differentiation. Such changes may arise from an interaction between these processes, or more simply from intrinsic differences in proliferation rates among distinct cell types. Cell-cycle inhibition may disrupt these differences, thereby preferentially reducing the proportions of the rapidly cycling populations that may rely on proliferation to maintain their numbers. Differences in sensitivity to the inhibitor were also unlikely to simply reflect target expression, as the target kinases Cdk4 and Cdk6 were approximately uniformly expressed across developmental time points and lineages (Figure S3J).

To disentangle these effects, we sought to estimate how much of the observed differences in cell-type proportions could be explained by variation in proliferation rates in the different lineages. Yet, our understanding of how cell cycle speed varies between cell types during mammalian development is still limited by a lack of quantitative data. To address this gap, we used our previously published simplified inference of cell-cycle periods across cell populations from single-cell data^72^ (STAR Methods). This method relies on estimating temporal delays between pre-mRNA and mRNA expression profiles of rhythmic genes, using the previously identified cell cycle phase (Figure 4A). First, we validated the inferred phases by examining UMI counts, which increased along the cell cycle, consistent with the expected cellular growth over one cell cycle (Figure 4B). Likewise, histone transcripts exhibited a distinct peak during S phase, consistent with DNA replication. Moreover, early progenitor cells displayed an enrichment in S/G2/M phases and a marked depletion in G1, consistent with their characteristically shorter G1 phase. Using harmonic regression (STAR Methods), genes that exhibited rhythmic expression, defined by a sufficient amplitude in both spliced and unspliced counts, were identified. The genes selected on this basis were largely known cell cycle regulators, and their inferred peak phases were consistent with established phase annotations (Figure 4C). Consistent positive shifts were observed between unspliced and spliced counts, reflecting the temporal ordering in which unspliced RNA precedes spliced RNA (Figure 4D; Figures S4A and S4B). The distribution of these shifts across different cell groups was used to estimate the relative cell cycle periods (relative to the half-lives of the genes, STAR Methods).

**Figure 4:**
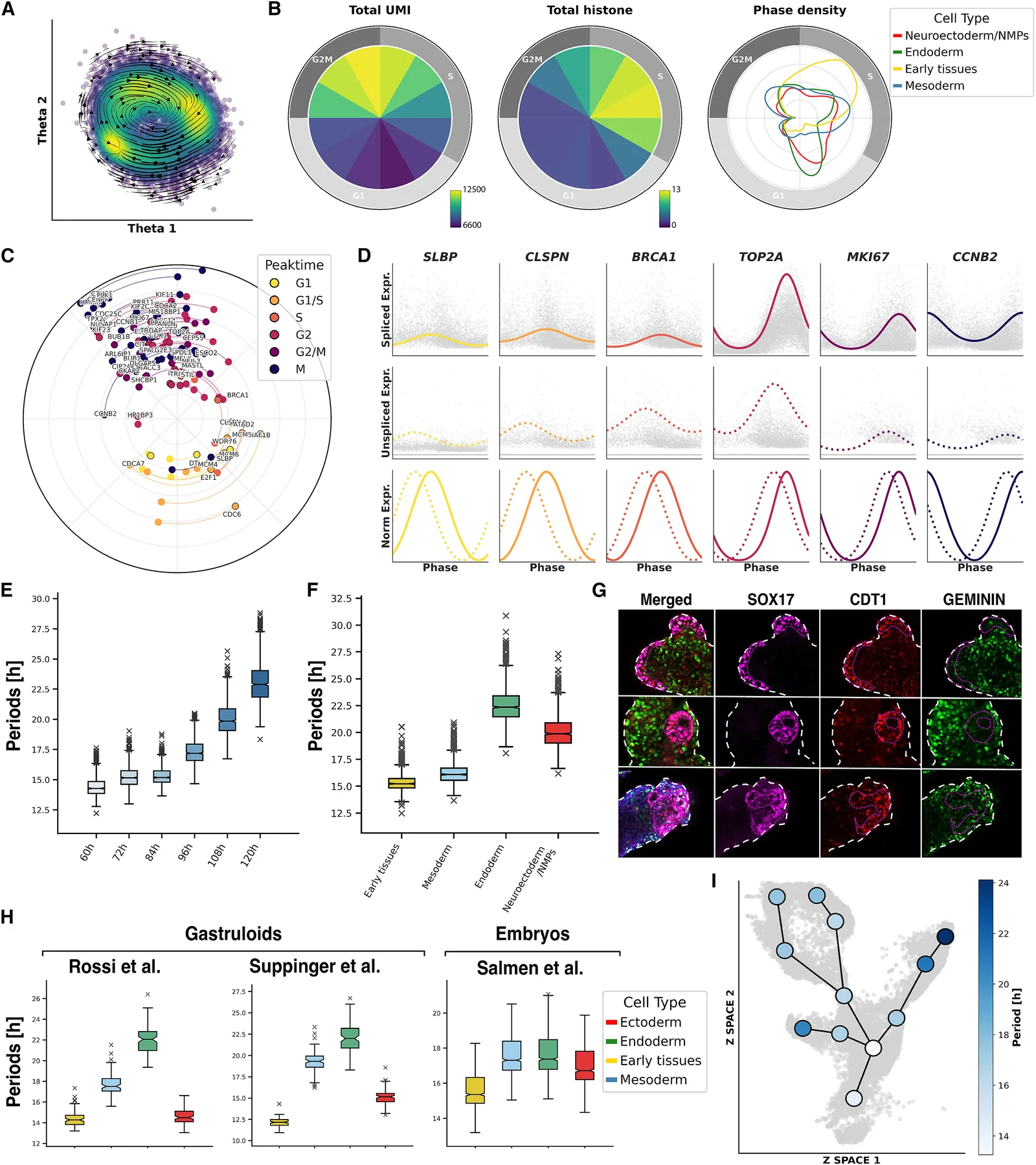
Unspliced-to-spliced RNA shifts allows mapping of cell cycle periods across cell types (A) The variational autoencoder infers cell cycle phases from scRNA-seq data within a *θ* space, where RNA velocity trajectories are subsequently projected. (B) UMI and histone profiles across cell-cycle phases validate phase assignment, with UMI counts doubling during replication and histone expression peaking in S phase. Phase distribution across cell types shows early progenitors are mostly in S and G2M phases. (C) Unspliced and spliced (black outline) transcripts are plotted by their peak phase (angle) and amplitude (radius), linked by arcs; colors indicate annotation of cell-cycle phases from *Cyclebase3*.^73^ (D) Cell-cycle gene profiles reveal systematic phase shifts between unspliced and spliced RNA. (E) Inferred periods show cell cycle lengthening during differentiation. (F) Velocity analysis across cell types reveals longer cycles in endoderm. (G) Fucci gastruloids confirm an overrepresentation of G1 cells in endodermal lineages. (H) Similar cycle length ratios are observed in other public datasets. (I) Finer temporal binning enables mapping of cell-cycle periods along differentiation trajectories. All boxplots show median, 25th/75th percentiles (box), and 1.5× IQR whiskers.

As an initial validation, this analysis was first applied to different differentiation stages, revealing a progressive lengthening of the cell cycle over time consistent with the expected slowdown of proliferation during differentiation (Figure 4E; Figure S4C). Extending the analysis to the cell types revealed distinct proliferation rates across lineages, with endodermal lineages exhibiting slower cycles (Figure 4F; Figure S4D). The slower proliferation of endodermal cells was also reflected in an enrichment of G1-phase cells, apparent both in the *θ*-space phase (Figure 4B, right) and in Fucci imaging of gastruloids, where Sox17-positive regions were predominantly in G1 (Figure 4G). The relative cell cycle periods were further validated using publicly available gastruloid and embryo datasets (Figures S4E–S4I″). In all cases, the inferred periods increased with differentiation, and the relative proliferation patterns were consistent, with ectoderm cycling faster than mesoderm and endoderm (Figure 4H). To characterize how cell cycle dynamics progress along differentiation, period inference was performed at successive nodes along the trajectories, yielding a continuous landscape of generally slowing down proliferation rates along the lineages (Figure 4I).

To assess whether the differences in proliferation rates inferred under control conditions could by themselves explain the changes in cell-type proportions observed after CDK4/6 inhibition, we constructed a simple population dynamics model. In this framework, the trajectories were binned into nodes, each representing a segment of the differentiation continuum through which cells progress over time (Figure S5A). The model captures how cell proportions evolve along these nodes by integrating two key processes: proliferation within each node, defined by node *i*-specific growth rates (*g*_*i*_), and differentiation between node *i* and *j*, described by transition rates (*W*_*ij*_) (Figure 5A). Differentiation transitions were restricted to biologically adjacent nodes, reflecting known lineage progressions (Figure 5B). Growth rates (*g*_*i*_) were fixed to the values inferred from the RNA-velocity model, while the transition rates (*W*_*ij*_) were fitted to recapitulate the cell proportions observed under control conditions (Figure S5B; STAR Methods). This provided a baseline model capturing normal proliferation-differentiation dynamics. To assess the impact of cell-cycle perturbation, a second model was simulated assuming null growth from the time of treatment (72 h), consistent with the approximately constant cell number observed experimentally under palbociclib treatment (Figure S2E). In this second model, the transition rates were kept constant and taken from the baseline model, while all growth rate values were set to zero from 72 h onward, allowing quantification of changes in cell proportions driven solely by the absence of proliferation, independent of changes in the differentiation rates.

**Figure 5:**
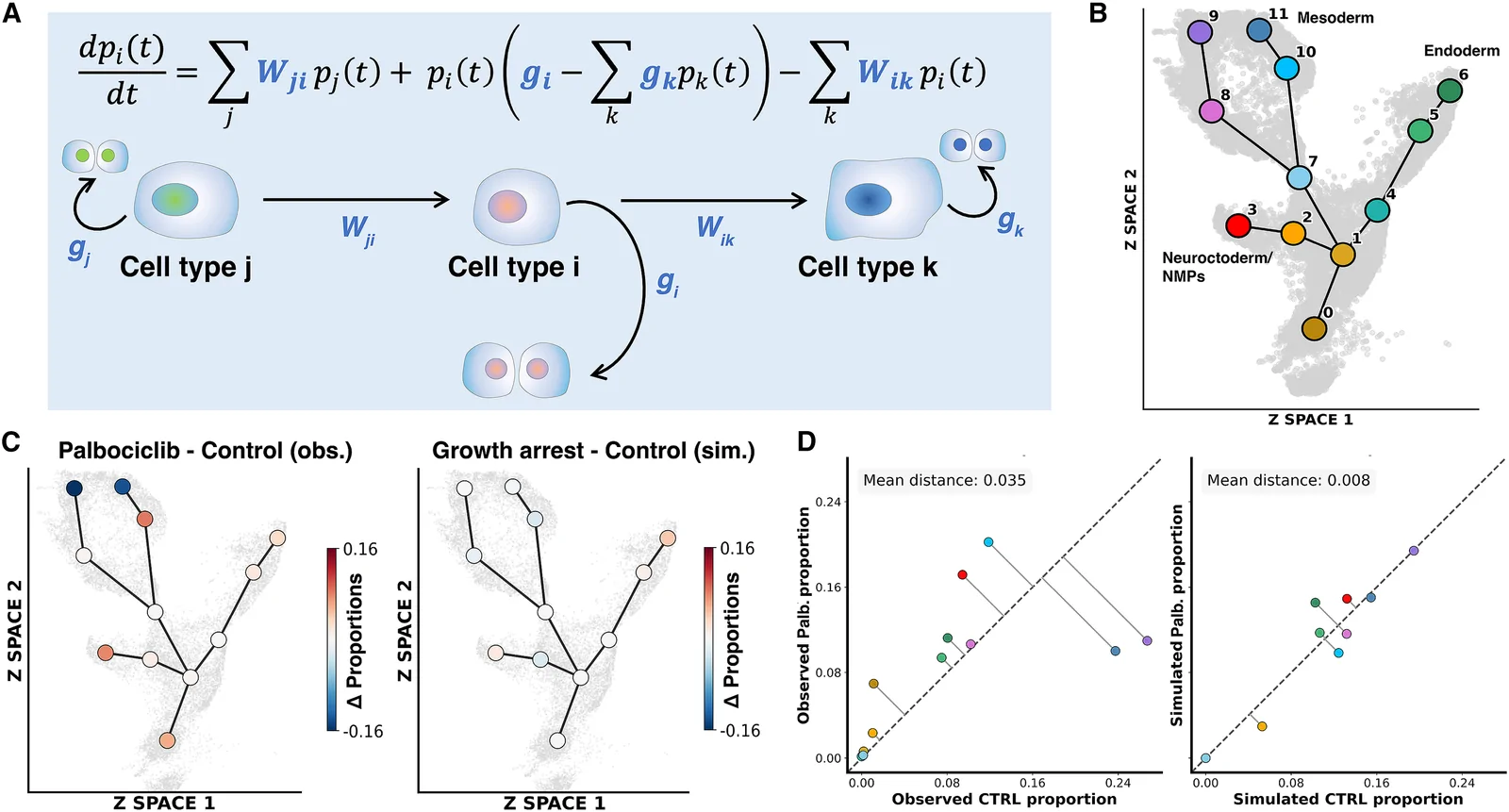
Population modeling suggests that cell cycle rate differences cannot be the sole cause of CDK4/6-induced cell type rebalance (A) Schematic of the population dynamics model with growth (*g*_*i*_) and differentiation (*W*_*ij*_) rates governing cell type proportions. (B) Cell clusters were defined as nodes along the lineage trajectories of the *z*-space, for which population densities were modeled over time. (C) Comparison of observed and simulated cell type shifts. CDK4/6 inhibition and growth arrest simulations show changes in the same direction, but CDK4/6 inhibition causes much larger shifts. (D) Scatterplot showing that deviations from the *x* = *y* line are approximately 4-fold larger for CDK4/6 inhibition than for growth arrest simulations.

The growth arrest simulation predicted only minor changes in cell-type proportions, indicating that the cell proportions are intrinsically robust to the loss of proliferation alone (Figures 5C and 5D). When compared with experimental data, both simulated and observed trends pointed in the same direction. Slow-cycling lineages such as endoderm were enriched, while fast-cycling tissues like mesoderm were reduced in proportion, consistent with our experimental observations under CDK4/6 inhibition. However, the magnitude of the changes in proportions under CDK4/6 inhibition was approximately 4-fold greater than expected from growth arrest alone (Figure 5D). Furthermore, the persistence of undifferentiated populations and the delayed progression of differentiation observed in the experimental data of treated gastruloids could not be explained by proliferation differences. Together, these results suggest that intrinsic, lineage-specific cell cycle dynamics account for part of the observed shifts, but additional cell-cycle-dependent mechanisms also alter specific differentiation rates and thus bias cell fate decisions, collectively shaping the full phenotypes seen in palbociclib-treated gastruloids.

## Discussion

Here, we used mouse gastruloids to uncover the developmental consequences of cell-cycle inhibition. We found that, despite near-complete growth arrest, key morphogenetic events such as symmetry breaking, elongation, and germ-layer patterning proceed largely unperturbed, demonstrating a remarkable robustness of core developmental programmes to proliferation perturbations. Nevertheless, microscopy, single-cell and whole-gastruloid transcriptomics revealed consistent shifts in cell type proportions, most notably a reduction in mesodermal lineages and signs of delayed differentiation. By inferring proliferation rates from scRNA-seq data, we show that these changes are only partly explained by cell-type-specific cell cycle kinetics, indicating that cell-cycle perturbations also modulate differentiation timing and lineage allocation more directly.

### Robustness of early development under cell-cycle perturbations

A central finding is that gastruloids exposed to CDK4/6 inhibition, although up to 9-fold smaller, remain capable of symmetry breaking, axis formation, elongation, and patterning, underscoring strong developmental robustness. Similar robustness to proliferation arrest has been reported in zebrafish,^46^^,^^47^^,^^48^ *C. elegans*,^42^ and *Xenopus*,^43^^,^^44^^,^^45^ despite some cell type-specific defects.^41^

However, the consequences of cell-cycle inhibition on mammalian development have only been sparsely characterized. Although mouse development appears largely robust to knockouts of cell cycle regulators,^49^^,^^50^^,^^51^ this does not imply robustness to changes in cell cycle dynamics; rather, it suggests that cell cycle dynamics themselves are robust to the loss of these regulators. For instance, in CDK4/6 double-knockout embryos, which undergo largely normal organogenesis with appropriate cell type proportions yet die later in development due to defective hematopoiesis, cell cycle dynamics are only minimally affected, as reflected by a modest size reduction and normal BrdU incorporation.^74^ Compared with the minimal *in vivo* response, palbociclib-treated gastruloids show a strong cell cycle response, including up to a 9-fold size reduction, reduced EdU incorporation, and increased G1 occupancy, providing a unique system to examine how real perturbations of cell cycle dynamics influence early developmental programs, a question previously addressed only in specific lineages^53^^,^^54^ or later stages.^40^

### Cell cycle dynamics as a regulator of lineage balance

In this study, we also observed a systematic rebalancing of cell type proportions under cell cycle inhibition, confirmed across imaging quantifications, individual gastruloid transcriptomes, and single-cell sequencing data. These shifts in proportions were approximately at most 2-fold, so five times less important than those reported in zebrafish embryos under similar experimental designs,^48^ suggesting greater robustness of cell type proportions in mouse gastruloids. However, differences in the timing of inhibition and in the rates of both cell-cycle progression and differentiation between the species may limit the direct comparability of these studies.

Nevertheless, these differences raise the question of whether the cell cycle acts as a regulatory mechanism to establish specific cell type proportions through lineage-dependent dynamics. This notion is closely related to the classical distinction between mosaic and regulative modes of development. In mosaic development, cell fate is largely predetermined by intrinsic factors,^75^ whereas in regulative development, fate depends on positional cues and cell interactions.^76^ In the context of cell-cycle arrest, the question becomes whether cell types proliferate at specific rates to establish the correct proportions, or whether these proportions are set regulatively, independent of lineage proliferation. In the literature, several studies support a role for lineage-specific proliferation in regulating cell type proportions. In zebrafish, fast-cycling lineages are selectively reduced upon cell-cycle arrest.^48^ Moreover, a recent study demonstrated the importance of apoptosis-mediated cell competition in the gastruloid system, highlighting the role of overall growth rates in shaping cell population dynamics.^69^

Assessing this effect requires first understanding how cell-cycle speeds vary between emerging cell types. Therefore, in this study, we propose a new approach to map cell type-specific cell-cycle dynamics along differentiation trajectories (Figure 4). This method is inspired by a recent development in RNA velocity that leverages unspliced and spliced RNA counts of rhythmic genes to infer cell-cycle periods across different cell populations, and whose accuracy has been validated across multiple datasets.^72^ While in this study we further validate the approach using complementary analyses (Figure 4; Figure S4), we acknowledge that the estimates obtained are only relative to the mean half-life of the cyclic transcripts.

Using the inferred cell cycle rates, we found that fast-proliferating cell types are indeed the most reduced after cell-cycle inhibition, suggesting that cell cycle kinetics may partly contribute to the observed shifts in cell type proportions. However, simulations using a minimal model of cell growth and differentiation showed that even in the scenario where proliferation rate differences between cell types were removed, this factor alone could not explain the proportional rebalancing observed under CDK4/6 inhibition (Figure 5). Together, these findings suggest that cell type specific cell cycle tuning affects, but does not solely determine cell type proportions differences under an inhibited cell cycle.

### Mechanisms of cell cycle and cell-fate decision coupling

So what other mechanisms could explain the rebalancing of cell type proportions that we observe? Different mechanisms of cell cycle gating of cell-fate decisions have been recently reviewed.^41^ For example, several studies have reported direct coupling between cell cycle regulators or cell-cycle phase distribution and cell fate commitment. Notably, inhibition of CDK4/6 or CDK2 in simpler 2D cellular models, including through palbociclib treatment, has been shown to impair mesoderm specification, primarily by reducing T expression.^27^ Additional studies have reported that palbociclib can promote neuronal differentiation.^30^ Moreover, CDK4/6 activity has been directly shown to suppress endoderm differentiation via the Smad2/Smad3 pathway.^34^ Altogether, these findings support a model in which CDK4/6 inhibition favors ectodermal and endodermal fates while reducing mesodermal contributions, which is consistent with our observations. While additional experiments are needed to elucidate the mechanisms involved, the previous study reporting mesodermal impairment had ruled out downregulation of BMP, WNT, or FGF signaling under CDK4/6 inhibition, suggesting that alternative pathways may be at play.^27^ One possible mechanism could involve cell cycle regulators-mediated remodeling of chromatin structure, as has been reported previously.^25^^,^^26^^,^^77^

Another possibility we cannot fully exclude is that the inhibitor exerts off-target effects directly on differentiation regulators, in addition to its specific impact on the cell cycle. For example, palbociclib has reported CDK4/6-independent effects, including modulation of proteasome activity, DNA replication, topoisomerase II, PRMT5 activity, and MDM4 splicing, which could potentially contribute to the altered differentiation phenotypes observed here.^78^^,^^79^^,^^80^ Moreover, the study reporting mesodermal impairment under cell-cycle arrest also found consistent effects with CDK2 inhibitors and genetic manipulations, including conditional gene knockdowns.^27^

Finally, our findings also highlighted a delayed differentiation under cell cycle-inhibited conditions, characterized by a population that failed to differentiate into any germ layer, as well as a delay within each germ layer, particularly in the mesoderm lineage. This observation contrasts with studies in pluripotent stem cells reporting that inhibition of cell cycle regulators can promote differentiation, suggesting an inverse relationship between proliferation and lineage commitment timing.^18^^,^^19^^,^^20^^,^^21^^,^^22^^,^^23^ *In vivo*, premature G1/S restriction point has similarly been shown to trigger early neuronal differentiation and disrupt neural tube formation.^53^^,^^54^ In contrast, other studies have reported effects consistent with our observations, where cell cycle inhibition led to reduced differentiation efficiency, notably within mesodermal derivatives.^27^ A global differentiation delay has also been observed *in vivo* at the whole-embryo level in zebrafish. Together, these findings support a context-dependent role of cell cycle regulation in controlling differentiation timing. A possible mechanism underlying the delayed differentiation aligns with some previously proposed models,^34^ in which cells must reach a permissive phase of the cell cycle to initiate differentiation. A globally slowed cycle could hinder progression to this phase in a fraction of cells, thereby delaying differentiation onset.

### Developmental scaling with gastruloid size reduction

Independently of coupling between cell cycle and cell-fate decision, the observation that the resulting cell cycle inhibited gastruloids are considerably smaller than control ones raises a broader question of scalability, that is, how the system preserves proportionality in geometric properties, cell type composition, and patterning despite variations in overall size. Such scaling is far from trivial, as gastruloid development is governed by processes such as cell sorting and tissue rearrangements, which are also inherently linked to the total number of cells within the system. This phenomenon has been investigated across species^81^ and, more recently, explored in gastruloid models,^65^^,^^82^ where varying the initial cell seeding number produced gastruloids spanning a wide size range. Although the authors observed systematic, size-dependent timing differences of spatial events, such as delayed symmetry breaking in larger gastruloids, the overall transcriptomic profiles remained robust across sizes. These findings suggest that the transcriptomic differences observed in our case cannot be attributed to size variation, independent of cell cycle effects. Furthermore, the absence of detectable delays in symmetry breaking in our system may reflect the fact that the pronounced size differences emerge only after this developmental event has already occurred.

In summary, this study provides new insights into how early mammalian development responds to cell-cycle perturbations. By combining imaging, transcriptomics, and modeling, we show that key developmental processes remain largely robust to near growth arrest, while lineage balance and differentiation timing are systematically altered across data modalities. Our work also introduces an approach to infer cell type-specific proliferation dynamics and highlights other potential interplay between cell-cycle regulation and cell-fate commitment. Together, these findings refine our understanding of the interplay between proliferation and development of self-organizing mammalian systems.

### Limitations of the study

Several limitations should be considered when interpreting these findings. First, gastruloids represent a simplified, self-organizing model of mammalian development and do not fully recapitulate the embryonic environment. In particular, they lack extraembryonic tissues and some embryo-specific structures and therefore do not reproduce all of the signaling, mechanical constraints, and spatially organized morphogen inputs that may couple proliferation to cell-fate decisions *in vivo*. Consequently, the robustness to growth arrest observed here should not be assumed to extend directly to intact embryos or to later developmental stages. Second, our mechanistic analyses rely primarily on pharmacological CDK4/6 inhibition with palbociclib. Although CDK4/6 target engagement was confirmed and similar phenotypes were observed with the structurally distinct CDK4/6 inhibitors ribociclib and abemaciclib, as well as related trends following CDK2 inhibition, CDK4/6-independent effects of palbociclib or lineage-specific effects on cell survival cannot be fully excluded. Temporally controlled genetic perturbations would therefore provide an important future validation of the causal contribution of CDK4/6 activity but establishing a suitable CDK4/6-deficient ES-cell system with sufficient temporal control remains technically challenging in gastruloids. Third, the scRNA-seq experiments were performed on pooled gastruloids, enabling robust characterization of population-level developmental trajectories but preventing direct quantification of gastruloid-to-gastruloid variability. Future studies combining individual-gastruloid single-cell profiling, temporally controlled genetic perturbations, and more integrated embryo models will therefore be important to determine how broadly the coupling between cell-cycle dynamics and lineage balance observed here applies to mammalian development *in vivo*.

## Resource availability

### Lead contact

Requests for further information and resources should be directed to and will be fulfilled by the lead contact, Felix Naef (felix.naef@epfl.ch).

### Materials availability

Reporter cell lines generated in this study are available from the lead contact.

### Data and code availability

• Bulk RNA-seq data (GEO: GSE311169; https://www.ncbi.nlm.nih.gov/geo/query/acc.cgi?acc=GSE311169) and single-cell RNA-seq data (GEO: GSE312366; https://www.ncbi.nlm.nih.gov/geo/query/acc.cgi?acc=GSE312366) have been deposited in the Gene Expression Omnibus and are publicly available.
• All original code has been deposited on GitHub (https://github.com/maleonardi/Leonardi_et_al_2026) and archived at Zenodo (https://doi.org/10.5281/zenodo.23317378), and is publicly available as of the date of publication.
• Any additional information required to reanalyze the data reported in this paper is available from the lead contact upon request.

## Acknowledgments

We thank members of the Naef group for discussions, particularly Cédric Gobet for his feedback on the manuscript and his help in publishing the transcriptomics data. The CGR8 and Sox1/T reporter mouse ES cell line were kindly provided by the Suter lab at EPFL. We thank the Gene Expression Core Facility, the BioImaging and Optics Platform and the Flow Cytometry Core Facility of EPFL for their support. This work was funded by the 10.13039/501100001703École Polytechnique Fédérale de Lausanne (EPFL).

## Author contributions

M.L. and F.N. conceived the project; M.L. designed, planned, and performed experiments. M.L. performed the computational analyses; Y.P. developed the variational auto-encoder for cell cycle phase assignment; M.L. wrote the manuscript with input from all authors. F.N. supervised the project.

## Declaration of interests

The authors declare no competing interests.

## Declaration of generative AI and AI-assisted technologies in the manuscript preparation process

During the preparation of this work, the authors used ChatGPT-5 to improve clarity, coherence, and linguistic quality of the text. After using this tool, the authors reviewed and edited the content as needed and take full responsibility for the content of the publication.

## STAR★Methods

### Key resources table

**Table:** 

REAGENT or RESOURCE	SOURCE	IDENTIFIER
**Antibodies**		

Goat polyclonal antibody anti SOX2	Santa Cruz Biotechnology	sc17320; RRID: AB_2286684
Rabbit monoclonal antibody anti T/BRACHYURY	Abcam	EPR18113; RRID: AB_2750925
Mouse monoclonal antibody anti E-CAD	BD Transduction Laboratories	610181; RRID: AB_397580
Rabbit monoclonal antibody anti SOX17	Abcam	EPR20684; RRID: AB_2801385
Rabbit polyclonal antibody anti Cleaved Caspase 3	Cell Signaling	#9661; RRID: AB_2341188
Donkey anti-Goat IgG Alexa 488 nm	ThermoFisher Scientific	A11055; RRID: AB_2534102
Donkey anti-Mouse IgG Alexa 647 nm	ThermoFisher Scientific	A31571; RRID: AB_162542
Donkey anti-Rabbit IgG Alexa 568 nm	ThermoFisher Scientific	A10042; RRID: AB_2534017
Donkey anti-Rabbit IgG Alexa 647 nm	ThermoFisher Scientific	A31573; RRID: AB_2536183

**Bacterial and virus strains**		

NEB 5-alpha Competent E.coli	New England Biolabs	C2987H

**Chemicals, peptides, and recombinant proteins**		

DMEM + Glutamax	Gibco	35050038
Sodium pyruvate	Gibco	11360070
*Β* - mercaptoethanol	Gibco	31350010
Penicillin/streptomycin	Gibco	15140122
Glutamax	Gibco	35050061
MEM Non essential amino acids (MEM NEAA)	Gibco	11140050
EmbryoMax 0.1% Gelatin Solution	Millipore	ES-006
LIF	EPFL Protein Production and Structure Core Facility	N/A
GSK3 inhibitor (CHIR99021)	Calbiochem	F252917-06-9
MEK1/2 inhibitor (PD0325901)	Selleckchem	S1036
PBS	Gibco	10010023
ES certified FBS	Gibco	10439024
Accutase	Corning	15323609
DMEM/F12 medium	Gibco	31331093
Neurobasal medium	Gibco	21103049
N2 supplement	Gibco	17502048
B27 supplement	Gibco	17504044
RAPIClear 1.52	SUNJin Lan	RC152001
Palbociclib	AdipoGen Life Sciences	CDX-P0590
NU6102	AdipoGen Life Sciences	AG-CR1-0020
RO3306	AdipoGen Life Sciences	AG-CR1-3515
Torin1	Calbiochem	475991
Porcupine	Focus Biomolecules	FBM-10-4605
FBS	Gibco	A5256701
Triton *X*-100	Sigma Aldrich	93443
DAPI	Thermo Scientific	62248
Blasticidin	InvivoGen	ant-bl-05
Lipofectamine 3000	Invitrogen	L3000001

**Critical commercial assays**		

RNeasy Micro kit	Qiagen	74004
EdU proliferation kit	Abcam	ab222421

**Deposited data**		

Single cell RNA sequencing	This paper	GEO:GSE312366
Bulk RNA sequencing of individual gastruloid	This paper	GEO:GSE311169

**Experimental models: Cell lines**		

CGR8	Provided by the laboratory of David Suter	Cat#07032901; RRID:CVCL_3987
Sox1-GFP::Brachyury-mCherry (SBR)	Provided by the laboratory of David Suter	based on CGR8 cell line
CGR8 Fucci(CA)5/H2B-iRFP720	This paper	based on CGR8 cell line

**Recombinant DNA**		

FUCCI(CA)5	Addgene	#153521
piggyBac transferase plasmid	Provided by the Protein production and structure Core facility of EPFL	N/A
pLenti-H2B-iRFP720	Addgene	#128961
pMD2.G	Addgene, Laboratory of Didier Trono at EPFL	#12259
psPAX2	Addgene, Laboratory of Didier Trono at EPFL	#12260

**Software and algorithms**		

ImageJ/Fiji v2.16.0	Schneider et al.^83^	https://imagej.nih.gov/ij/
Ilastik v1.4.0	Berg et al.^84^	https://doi.org/10.1038/s41592-019-0582-9
Stardist v0.9.1	Schmidt et al.^85^	https://github.com/stardist/stardist
Python v3.12.11	Python Software Foundation	https://www.python.org
STAR solo v2.7.11b	Dobin et al.^86^	https://doi.org/10.1093/bioinformatics/bts635
pydeseq2 v0.4.0	Muzellec et al.^87^	https://pydeseq2.readthedocs.io/en/stable/
Scaden v1.1.2	Menden et al.^66^	https://scaden.readthedocs.io/en/latest/
Cellranger v7.2.0	10× Genomics	https://www.10xgenomics.com/support/software/cell-ranger/latest/release-notes/cr-release-notes
Velocyto v0.17.17	La Manno et al.^88^	https://github.com/velocyto-team/velocyto.py
POT v0.9.5	Flamary et al.^89^	https://pythonot.github.io/
Scanpy v1.9.8	Wolf et al.^90^	https://scanpy.readthedocs.io/en/stable/
Scvelo v0.3.2	Bergen et al.^91^	https://scvelo.readthedocs.io/en/stable/
PyTorch v2.4.1	Paszke et al.^92^	https://pytorch.org/
Original analysis code	This paper	Zenodo: https://doi.org/10.5281/zenodo.23317378

### Experimental model and study participant details

#### Cell lines

This study used mouse (*Mus musculus*) embryonic stem (ES) cell lines; no animals or human participants were used. CGR8 ES cells (strain 129P2/Ola) and the CGR8-derived Sox1-GFP::Brachyury-mCherry (SBR) reporter line were provided by the laboratory of David Suter (EPFL). The CGR8-derived Fucci(CA)5/H2B-iRFP720 reporter line was generated in this study (see STAR Methods). All ES cell lines used in this study are of male origin. The influence of sex on the results could therefore not be assessed. Cell lines were tested negative for mycoplasma contamination.

### Method details

#### Cell culture

CGR8 mouse embryonic stem cells were maintained at 37°C in DMEM (*Gibco*, 61965059) supplemented with GlutaMAX (1×; *Gibco*, 35050038), MEM non-essential amino acids (1×; *Gibco*, 11140050), sodium pyruvate (1 mM; *Gibco*, 11360070), penicillin–streptomycin (1×; *Gibco*, 15140122), 2-mercaptoethanol (0.1 mM; *Gibco*, 31350010), and ES-qualified fetal bovine serum (10%; *Gibco*, 10439024). To maintain pluripotency, leukemia inhibitory factor (100 ng/mL; produced by the *EPFL* facility), GSK3 inhibitor (3 μM; *Calbiochem*, 252917-06-9), and MEK inhibitor (PD0325901; 1 μM; *Selleckchem*, S1036) were added to the culture medium. Cells were plated on gelatin-coated plates. Cells were passaged regularly to prevent confluency and spontaneous differentiation.

#### Establishment of cell reporter cell lines

CGR8 embryonic stem cells expressing Sox1-GFP/T-Bra-mCherry (SBR reporter line) were kindly provided by Cédric Deluz from David Suter’s laboratory.

To generate the Fucci-expressing line, CGR8 ES cells were transfected with the Fucci(CA)5 (*Addgene*: #153521) plasmid using the PiggyBac transposon system, as previously described.^93^ The Fucci constructs and the PiggyBac transposase were co-transfected at a 3:1 ratio using *Lipofectamine*. Transfected cells were selected with *blasticidin* (7 days), and individual Fucci-positive single-cell clones were subsequently isolated by fluorescence-activated cell sorting (FACS). In addition, a nuclear reporter was introduced using lentiviral transduction. Lentiviral vectors containing the H2B-iRFP fusion sequence (*Addgene*: #128961) were used in a second-generation lentiviral system. *HEK293T* cells were co-transfected with the lentiviral vector carrying the gene of interest and two packaging plasmids (pMD2.G and psPAX2, obtained from the *Trono* lab), using *HEPES* and *HeBS2X* medium. Viral particles were collected and concentrated 48 h after transfection. CGR8 ES cells were then infected with the produced lentivirus, and H2B-iRFP-positive clones were isolated by FACS.

#### Gastruloid generation

To generate gastruloids, we followed the original protocol described by Van den Brink et al.^59^ ES cells were resuspended in N2B27 medium composed of 50% DMEM/F12 (*Gibco*, 31331093) and 50% Neurobasal medium (*Gibco*, 21103049), supplemented with GlutaMAX (1×; *Gibco*, 35050061), MEM non-essential amino acids (1×; *Gibco*, 11140050), sodium pyruvate (1×; *Gibco*, 11360070), penicillin-streptomycin (1×; *Gibco*, 15140122), 2-mercaptoethanol (0.1 mM; *Gibco*, 31350010), N2 supplement (1×; *Gibco*, 17502048), and B27 supplement (1×; *Gibco*, 17504044). Cells were seeded into 96-well round-bottom plates at a density of 300 cells per well in 40 μL of medium. After 48h of incubation at 37°C, a 24h GSK3 inhibitor pulse (*Calbiochem*, 252917-06-9) was applied by adding 150 μL of N2B27 medium containing GSK3i (final concentration, 1×) to each well. After 24h (72h post-seeding), the medium was replaced with fresh N2B27, which was subsequently renewed every 24h.

#### Inhibitors

In this work, we used the various cell cycle and differentiation inhibitors listed below. For the CDK4/6 inhibitor, unless otherwise stated in the manuscript, it was applied at a concentration of 5 μM, starting 72h after aggregation.

**Table:** 

Inhibitor name	Inhibitor target	Reference
Palbociclib	CDK4/6 inhibitor	*AdipoGen Life Sciences*, CDX-P0590
NU6102	CDK2 inhibitor	*AdipoGen Life Sciences*, AG-CR1-0020
RO3306	CDK1 inhibitor	*AdipoGen Life Sciences*, AG-CR1-3515
Torin1	mTOR inhibitor	*Calbiochem*, 475991
Porcupine	Wnt inhibitor	*Focus Biomolecules*, FBM-10-4605

#### Immunofluorescence (IF)

For immunofluorescence assays, gastruloids were collected fixed in PFA 4% for 1 h at room temperature (RT). Gastruloids were then stored in PBS 1× at 4°C. The immunofluorescences were done as follows: After gastruloids were washed 3× in PBS and 3× in PBFST (PBS 1×, 10% FBS, Triton *X*-100 (0.5%)), each for 10min at RT, nonspecific binding of the primary antibody was prevented by a 1-h blocking step in PBSFT at 4°C. Subsequently, samples were incubated overnight at 4°C in the primary antibodies solution. The different antibodies used, their species and concentrations are below. Gastruloids were washed several times at 4°C in PBSFT (2 × 5 min, 3 × 15min and 4 × 1 h) and incubated overnight at 4°C in a PBFST solution with DAPI (*Invitrogen*™ 62248, 1:500) and the associated secondary antibodies. Gastruloids were then washed again in PBFST (3 × 20min), and then cleared using the RapiClear 1.52 clearing solution (*SUNjin lab*, #RC152002).

**Table:** Primary antibodies

Antibody target	Species	Reference	Concentration
Sox2	Goat polyclonal	*Santa Cruz Biotechnology*, sc17320	1:200
T-Bra	Rabbit monoclonal	*Abcam*, EPR18113	1:200
ECad	Mouse monoclonal	*BD Transduction Laboratories*, 610181	1:100
Sox17	Rabbit monoclonal	*Abcam*, EPR20684	1:200
Cleaved caspase 3	Rabbit polyclonal	*Cell Signaling*, #9661	1:400

**Table:** Secondary antibodies

Antibody target	Species	Fluorophore	Reference	Concentration
Mouse	Donkey	Alexa 647	*ThermoFisher Scientific*, A31571	1:500
Rabbit	Donkey	Alexa 538	*ThermoFisher Scientific*, A10042	1:500
Goat	Donkey	Alexa 488	*ThermoFisher Scientific*, A11055	1:500

#### EdU assays

The EdU assay was performed using the Proliferation Kit (*Abcam*, ab222421) according to the manufacturer’s instructions. At 118 h after aggregation, half of the medium of the gastruloids (75 μL per well) in control and CDK conditions was replaced with fresh medium (with or without the inhibitor) containing 20 μM EdU, resulting in a final concentration of 10 μM EdU in each well. Gastruloids were incubated with EdU for 2 h until the 120h endpoint. For microscopy, gastruloids were fixed with 4% PFA for 1 h. For flow cytometry analysis, gastruloids were collected, washed twice with PBS, and dissociated with *Accutase* for 5 min at 37°C. Cells were harvested, washed twice with washing buffer, and fixed in 4% PFA for 20 min.

Both fixed gastruloids and dissociated cells were washed twice with a washing buffer and permeabilized using the permeabilization buffer (30 min for single cells, 1 h for gastruloids). For dissociated single-cell samples, each centrifugation step was performed at 300 × g for 5 min. Cells and gastruloids were then washed twice, incubated with the reaction mix for EdU detection as described in the manufacturer’s protocol, and stained with DAPI (1:500). Incubation was carried out for 30 min for single cells and 1 h for gastruloids. After two final washes, cells were analyzed by flow cytometry, and gastruloids were cleared using the RapiClear 1.52 clearing solution (*SUNjin Lab*, #RC152002) before imaging.

#### Flow cytometry

Flow cytometry measurements (EdU and FUCCI assays) were performed on a *BD LSRFortessa*™ equipped with four lasers (405 nm, 488 nm, 561 nm, and 640 nm). Instrument settings were optimised using unstained controls. Cells were manually gated to exclude debris based on forward and side scatter density plots, and positivity thresholds were determined from fluorescence intensity histograms and negative controls.

#### Bulk BRB-seq library preparation of individual gastruloids

Gastruloids were grown from CGR8 cells according to the previously described protocol,^59^ with drug treatments applied from 72h onwards: Control (DMSO), CDK4/6 inhibitor (D1: 5 μM), CDK2 inhibitor (D1: 10 μM, D2: 20 μM), and mTOR inhibitor (D1: 1 μM, D2: 2 μM). For each condition, at least four gastruloids were collected at 120 h A.A. from the outer wells of the plate. RNA was extracted from single gastruloids using the *Qiagen RNeasy Micro Kit* (cat. no. 74004). Whole gastruloids were lysed in RLT buffer, and the lysate was homogenized using a *QIAshredder* (*Qiagen*, cat. no. 79656). The homogenized lysate was then passed through an RNeasy MinElute spin column to capture RNA. After assessing RNA quality on the TapeStation, RNA libraries were prepared using the *BRB-seq* protocol (*Alithea*).^94^ Libraries were subsequently sequenced using a 10XG run on the *NovaSeq* platform, targeting approximately 400 million reads.

#### scRNA-seq library generation

For single-cell RNA-seq (scRNA-seq) experiments, multiple batches of gastruloids were generated in parallel from the SBR reporter line, as described below (Figure S6). From 72 h onward, gastruloids were treated with either DMSO (control), *Palbociclib* (5 μM), or the *Porcupine* inhibitor (3 μM). At the collection time point, gastruloids from the external wells of a 96-well plate were harvested (approximately 40 gastruloids per condition). Samples were washed with PBS and dissociated using *Accutase* and pipetting. Following dissociation, cells were resuspended in PBS containing 0.04% BSA.

Each sample was labeled for 5 min according to the 10× Genomics Cell Multiplexing protocol (*Chromium Next GEM Single Cell 3′ v3.1 with Cell Multiplexing*). After three washes in PBS with 1% BSA, cells from different conditions were counted and pooled. The pooled suspension was filtered twice using 40 μm cell strainers (*Sigma*, BAH136800040-50 EA) to remove cell clusters and debris.

Single-cell suspensions were processed using the *10x Genomics Chromium* system to encapsulate individual cells in Gel Beads-in-Emulsion (GEMs) and barcode polyadenylated mRNA transcripts for downstream library preparation. Libraries were prepared following the manufacturer’s instructions and sequenced on an *Illumina NovaSeq 6000* platform, targeting approximately 800 million reads per run (100-cycle kit).

### Quantification and statistical analysis

#### Statistical analysis

Unless otherwise stated, *n* refers to the number of individual gastruloids (biological replicates); the exact value of *n* for each experiment is reported in the corresponding figure legend. Data are represented as mean ± SD, except in Figure S3F, where error bars were obtained by bootstrapping cells within the sample. Boxplots show the median (center line) and 25th/75th percentiles (box); whiskers extend either to 1.5× the interquartile range or to the 5th/95th percentiles (with all data points shown, jittered), as specified in each figure legend. Violin plots show the kernel density estimate of the distribution, with dashed lines indicating the 25th, 50th (median), and 75th percentiles. Statistical significance was assessed using a two-sided Welch’s *t* test (Figure 1F; Figures S2C and S2P, on log- or log-CPM-transformed data; Figures 3F and 3G; Figure S3I) or a two-sided Mann-Whitney U test (Figures S1F and S1H); significance of cell-type proportion fold-changes (Table S4) was assessed from 2,000 bootstrap resamples of the scRNA-seq dataset, with *p*-values corrected for multiple comparisons using the Bonferroni method. Significance thresholds are indicated in figures as *p* < 0.05 (∗), *p* < 0.01 (∗∗), *p* < 0.001 (∗∗∗), and, where indicated, *p* < 0.0001 (∗∗∗∗); Cohen’s *d* is reported as a measure of effect size where indicated. All statistical analyses were performed in Python.

#### Microscopy and image quantification

##### Brightfield live imaging

Brightfield imaging of live gastruloids over time (every 24h) was performed on the *PerkinElmer Operetta* microscope equipped with a 10×/0.35 objective. Gastruloids were segmented using pixel classification in *ilastik* software.^84^ Elongation was computed from binary object masks as the ratio of the Feret diameters measured along the major and minor axes, determined by principal component analysis (PCA) of the foreground pixel coordinates. For all conditions, gastruloids were imaged from the outer wells of the plate, which showed a consistently high frequency of elongation. No filtering based on elongation was subsequently applied; gastruloids were excluded only for technical reasons, including incomplete imaging, unsuitable orientation, or poor focus. Gastruloids were resuspended before imaging, but no specific procedure was used to control their orientation relative to the imaging plane.

##### Fluorescent confocal imaging and quantification

For cell-type marker immunofluorescence, Fucci gastruloids, caspase stainings and EdU stainings, gastruloids were imaged using a *Leica SP8* confocal microscope equipped with either a 16× or a 20× immersion objective.

To quantify the distribution of different cell types or caspase levels, each voxel of each z stack was classified as positive or negative for individual fluorescence channels (DAPI, T, Sox2, and E-cadherin) using intensity thresholding (thresholds were selected manually). Following thresholding, binary masks were generated for each marker and subjected to a median filter to reduce noise. The resulting masks were then combined to assess co-expression patterns between markers. Ratios of different cell populations were calculated as the number of pixels positive for a given marker (or marker combination) divided by the total number of DAPI-positive pixels.

Cell cycle phase quantification in Fucci gastruloids was performed as follows. Cells were segmented independently in each Z-slice using *StarDist*,^85^ first on the sum of the red and green fluorescence channels, and then separately on each channel. A cell was considered positive in the red or green channel if the centroid of the cell identified in the summed image also fell within a segmented region of the corresponding individual channel.

#### RNA-seq data analysis of the individual gastruloids

Raw reads from the BRB-seq experiment were demultiplexed and mapped using *Alithea BRB-SeqTools*. Differential gene expression analysis and CPM-based comparisons between samples were performed using the *DESeq2* library^87^^,^^95^ implemented in Python.

For cell-type deconvolution, the algorithm described in *Scaden* was applied following the authors’ instructions.^66^ The *Scaden* model was first trained on our own scRNA-seq dataset (control condition, 120h) using our cell-type annotations, and subsequently applied to the bulk RNA-seq expression matrix to estimate the proportion of each cell type.

#### scRNA-seq data analysis

Raw sequencing reads were mapped to the reference genome using *Cell Ranger*^96^ (10× Genomics). Spliced and unspliced transcript counts were then extracted and demultiplexed using the *Velocyto* pipeline.^88^ Data processing and quality control were carried out using the *Scanpy*^90^ library in Python. Cells with low total UMI counts or a high proportion of mitochondrial gene expression were filtered out to exclude low-quality or dying cells. Lowly expressed genes were also removed. The counts were then normalised per cell using total count normalisation and log-transformed for subsequent analysis.

For cell type assignment, cells were first clustered using the *Louvain* algorithm, after which cell type markers (list generated from refs.^64^^,^^97^) were used to manually annotate each cluster.

#### Estimation of latent cell state and cell cycle phases

From the scRNA-seq data, we estimate cell cycle phases for each individual cell, while accounting for context-specificity arising from biological factors such as cell type.

For this, we used a variational autoencoder (VAE), with a structured latent space consisting of a cell cycle phase (*θ*), and a cell-context variable *z*, as described in^71^ (GitHub repository: https://github.com/gityves/CoPhaser).

Briefly, the model^71^ uses as input a set of 98 cell cycle genes, and the 2000 most variable genes in the dataset. The variable genes are projected onto a ten-dimensional latent coordinate *z*, using the mean and variance of an isotropic multivariate Gaussian distribution. The cell cycle genes project onto a two-dimensional space from which the angle is modeled as a power spherical distribution.^98^ For each gene *g*, the latent variables are then decoded into the sum of an aperiodic component and a circular component consisting of a Fourier series.

#### scRNA-seq trajectory reconstruction

To reconstruct temporal trajectories of cells within the *z*-space, we employed optimal transport (OT) to link cell distributions between successive time points for each experimental condition, using the *Python Optimal Transport*^89^ library.

A common set of baseline cells at 0h was randomly selected to initialise representative trajectories. For each condition, cell coordinates at consecutive time points were treated as source and target distributions.

Pairwise squared Euclidean distances between all cells at time *t* and time *t*+1 were computed to form the transport cost matrix, and the Earth Mover’s Distance (EMD) solution was then obtained to derive the optimal transport plan that minimises the overall transport cost. This procedure was applied iteratively across all consecutive time intervals, yielding sets of synthetic trajectories that approximate the most probable flow of cell populations through the embedding over time.

The resulting trajectories were subsequently projected onto a lower-dimensional representation using principal component analysis (PCA) and clustered with k-means to group similar patterns. Each cluster corresponded to a putative lineage, which we annotated manually. Finally, we quantified and compared the number of trajectories in each cluster across conditions to reveal biases in lineage commitment and cell-state transitions.

For each trajectory cluster, we computed the mean trajectory, which served as the reference path for pseudotime assignment. For these mean trajectories, we calculated the shortest Euclidean distance from each cell’s position in *z*-space to the piecewise linear curve defined by the trajectory points; cells within a fixed distance threshold were considered associated with that trajectory.

For each associated cell, pseudotime represents how far along the trajectory the cell lies, relative to its start and end. To compute this, we measured the total length of the trajectory and the distance along the path from the start to the point on the trajectory closest to the cell (its projection). The pseudotime value is then this distance divided by the total trajectory length, giving a normalised value between 0 (trajectory start) and 1 (trajectory end).

#### Simplified RNA velocity model for cell cycle period inference

We here used our previous simplified RNA velocity model^72^ to estimate cell cycle periods across the lineages, which is based on the delays between mRNA and pre-mRNA accumulation profiles.

To characterise those delays genome-wide, we used the cell cycle phases (*θ*_*c*_) inferred from the VAE and fitted each gene with a harmonic regression (one harmonic) using a generalized linear model (GLM) with Poisson noise.

For each gene, expression levels across cells were modeled as a one-harmonic Fourier series, specifically the expected mean counts was taken as

(Equation 1) $$\lambda_{c}=L_{c}\;\exp\left(\beta_{0}+a\;\cos\left(\theta_{c}\right)+b\;\sin\left(\theta_{c}\right)\right)$$

where *L*_*c*_ denotes the total transcript number per cell (used as an offset) and [*β*_0_,*a*,*b*] are the gene specific regression coefficients to be estimated.

Model parameters were estimated for each gene individually using iteratively re-weighted least squares (IRLS).

The model was first applied genome-wide to the spliced transcripts to identify genes with clear periodic mRNA profile. Fitted log-amplitudes greater than 0.2 (log) were selected for further analysis. Subsequently, the same model was refitted on the unspliced transcript layer for the subset of oscillatory genes. Genes with unspliced amplitudes exceeding 0.1 (log) were kept for further analyses, to ensure periodic spliced and unspliced expression.

As in,^72^ the phase shifts Δ*ϕ*_*sg*_ between unspliced and spliced mRNA in condition s then depend on the transcript half-lives $\tau_{g}=\gamma_{g}^{-1}$ and frequencies *ω*_*s*_ = 2∗*π*/*T*_*s*_ (*T*_*s*_: period) according to

(Equation 2) $$\delta_{sg}=\tan\left(\Delta \phi_{sg}\right)=\omega_{s}\tau_{g}$$

To compare conditions, we selected on genes that were rhythmic in both spliced and unspliced counts across all conditions *s*.

This defines a rank-1 decomposition of the matrix *δ*_*sg*_. Using the singular value decomposition (SVD).

(Equation 3) $$\delta_{cg}=u_{c}\;d\;v_{g}+\text{higher}-\text{rank}\;\text{terms}$$

we can obtain condition-specific cell-cycle velocity

(Equation 4) $$\omega_{s}^{⋆}=u_{s}\;d\;\bar{v_{g}}$$

where $\bar{v_{g}}$ denotes the mean over genes. The corresponding cell-cycle period, expressed in units of mean transcript half-lives, is then given by

(Equation 5) $$T_{s}^{⋆}=\frac{2\pi }{\omega_{s}^{⋆}}\text{.}$$

For all analyses, the mean transcript half-life was fixed to *τ* = 1.25 h, consistent with typical mRNA decay times in mammalian single cells and with values reported in the literature, so as to yield cell-cycle periods within a biologically plausible range. Because inferred cell-cycle periods scale proportionally with (*τ*), changing this parameter would rescale the absolute period estimates but would not affect the relative differences or ratios between cell populations used in our analyses.

#### Cell type proportion model

Using the above trajectory reconstruction, we coarse-grained the cells into discrete nodes (11 nodes) along the different differentiation trajectories and for each inferred their growth rates using the simplified RNA velocity model. We also modeled biologically plausible transitions between nodes based on established biological knowledge.

To describe the temporal evolution of cell proportions across these nodes, we employed a linear ordinary differential equation (ODE) model capturing net cell growth and differentiation dynamics. The number of cells in node *i* at time *t*, denoted *N*_*i*_(*t*), evolves according to

(Equation 6) $$\frac{dN_{i}\left(t\right)}{dt}=g_{i}N_{i}\left(t\right)-\sum_{j}W_{ij}N_{i}\left(t\right)+\sum_{j}W_{ji}N_{j}\left(t\right)$$

where *g*_*i*_ represents the *effective growth rate* of node *i*, accounting for the combined effects of proliferation and apoptosis, and *W*_*ij*_ denotes the differentiation rate from node *i* to node *j*.

Because scRNA-seq data can only be used in relative rather than absolute cell numbers, we reformulated the model in terms of the *proportion* of cells in each node at time *t*:

(Equation 7) $$p_{i}\left(t\right)=\frac{N_{i}\left(t\right)}{N\left(t\right)},\text{with}\;N\left(t\right)=\sum_{i}N_{i}\left(t\right)$$

which yields the proportional model

(Equation 8) $$\frac{dp_{i}\left(t\right)}{dt}=p_{i}\left(t\right)\left(g_{i}-\sum_{k}g_{k}p_{k}\left(t\right)\right)-\sum_{j}W_{ij}p_{i}\left(t\right)+\sum_{j}W_{ji}p_{j}\left(t\right)$$

We note that the proportions are invariant with respect to a global shift in the growth rates (*g*_*i*_→*g*_*i*_-Δ).

We implemented the model in Python, solving the system of ODEs with the scipy package. The transition matrix (*W*) was fitted on the control condition data, after which we simulated a scenario of complete growth arrest by setting all node-specific growth rates to zero after 72 h while keeping the transition dynamics unchanged. The simulated trajectories were then compared to both the observed and fitted control trajectories to assess the impact of growth suppression on differentiation dynamics.
