# Supplementary material for: Cell-cycle inhibition preserves robust development but rebalances lineages in mouse gastruloids
Source: iScience. 2026 Nov 2;29(11):118084. doi: 10.1016/j.isci.2026.118084 (PMC13756060; doi:10.1016/j.isci.2026.118084)
Supplement: Supplementary file 1 [file mmc1.pdf]

iScience, Volume *29*

Supplemental information

Cell-cycle inhibition preserves robust development but rebalances lineages in mouse gastruloids

Maxine Leonardi, Yves Paychère, and Felix Naef

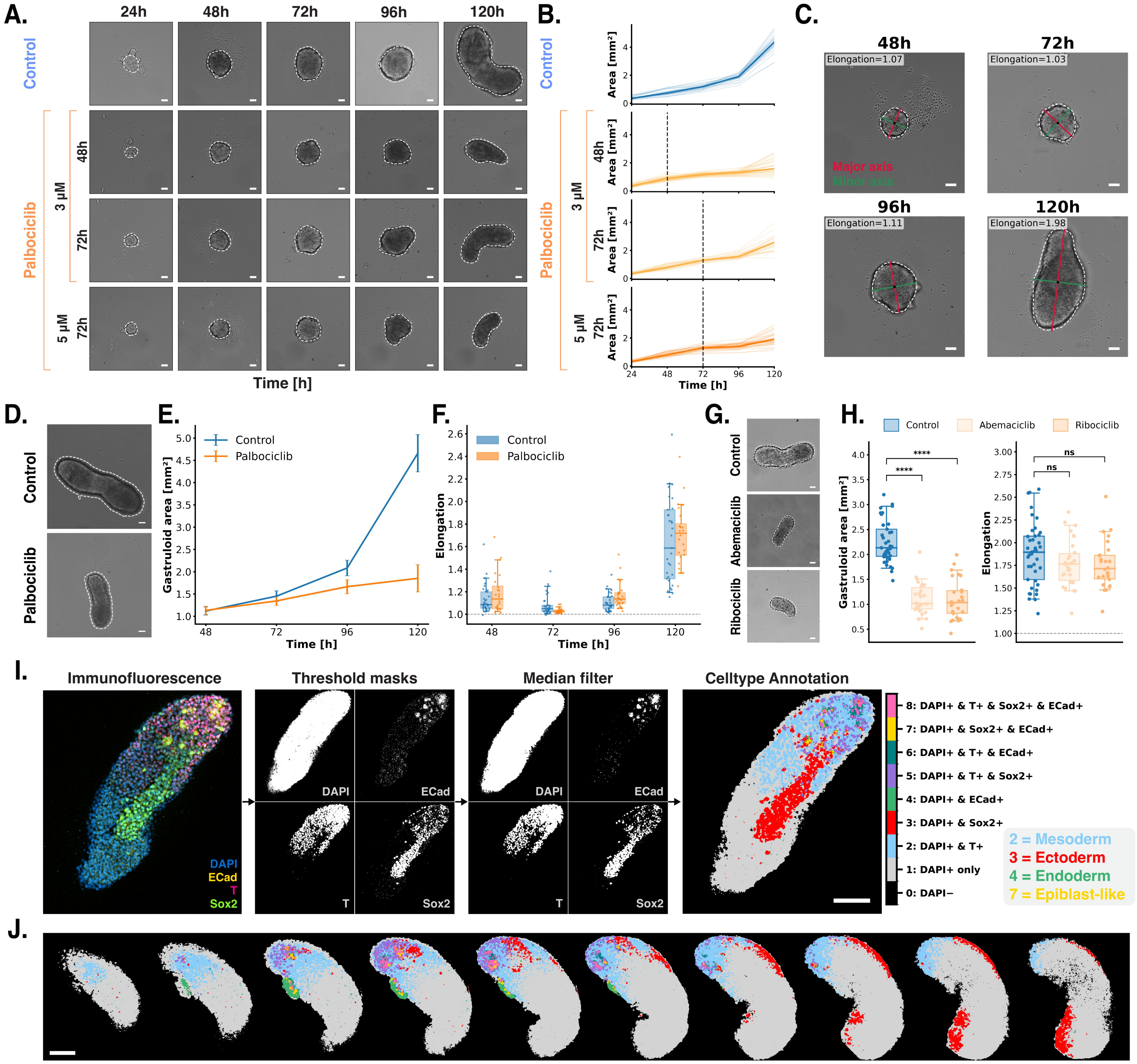

Figure S1. Quantification of gastruloid growth, elongation, and cell-type composition from microscopy images.

(A) Brightfield Operetta imaging tracks gastruloid morphology over time; white overlays show segmentation masks used for area quantification. Scale = 100 µm.

(B) Area progression curves of individual gastruloids over time demonstrate high size reproducibility within conditions. $n >= 20$ for all conditions.

(C) Elongation is quantified as the ratio of the major to minor axis, both fitted to the mask of gastruloid brightfield images. Scale = 100 µm.

(D) Representative images of E14-background gastruloids under control and Palbociclib-treated conditions. Scale = 100 µm.

(E) Area progression of E14-background gastruloids under control and Palbociclib-treated conditions, showing a significant reduction in growth following treatment. Mean $\pm$ SD.

(F) Quantification of elongation in E14-background gastruloids under control and Palbociclib-treated conditions, showing no significant difference between conditions.

(G) Representative images of gastruloids under control conditions and following treatment from 72 h with the CDK4/6 inhibitors Abemaciclib and Ribociclib. Scale = 100 µm.

(H) Quantification of gastruloid area and elongation following treatment from 72 h with Abemaciclib and Ribociclib, showing impaired growth but preserved elongation following CDK4/6 inhibition.

All box plots show median, 25th/75th percentiles (box), and whiskers extending to the 5th/95th percentiles; all data points are shown (jittered). Significance from a two-sided Mann-Whitney U test ($p < 0.05$ *, $p < 0.01$ **, $p < 0.001$ ***, $p < 0.0001$ ****).

(I) IF staining quantification pipeline. For each image z-stack, per-channel thresholds (DAPI, E-CAD, T, SOX2) classify pixels as positive or negative. A median filter removes noise. Pixels are then labelled by co-positivity: T-only = mesoderm; E-CAD-only = endoderm; SOX2-only = neuroectoderm/NMPs; SOX2+E-Cad = early tissues (epiblast-like). Scale = 100 µm.

(J) IF staining quantification is performed over the full z-stack for each gastruloid. Scale = 100 µm.

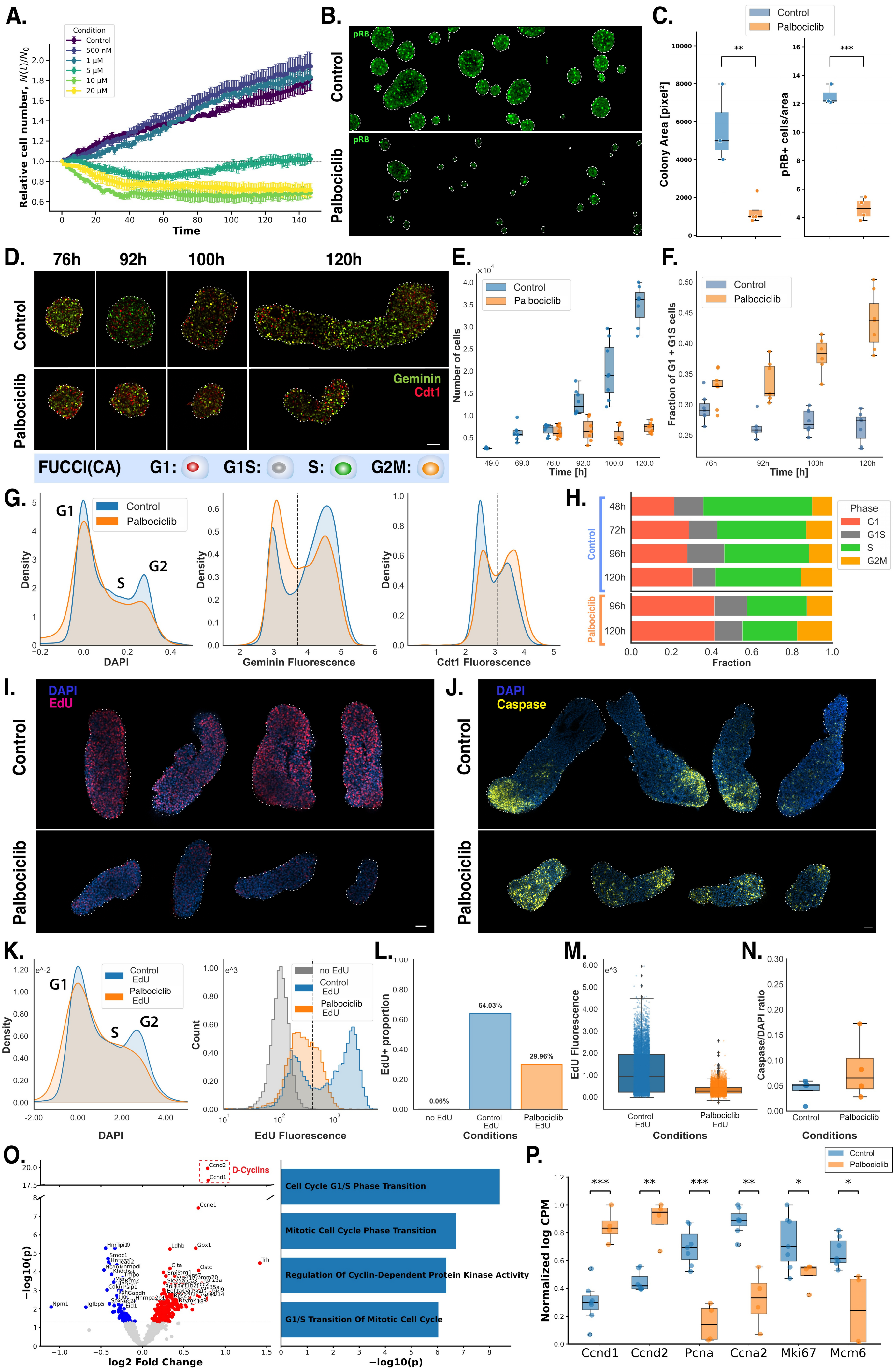

A.
Condition
Control
500 nM
1 µM
5 µM
10 µM
20 µM
Relative cell number, $N(t)/N_0$
Time
B.
pRB
Control
Palbociclib
C.
Control
Palbociclib
Colony Area [pixel²]
pRB+ cells/area
**
***
D.
76h
92h
100h
120h
Control
Palbociclib
Geminin
Cdt1
FUCCI(CA)
G1:
G1S:
S:
G2M:
E.
Control
Palbociclib
Number of cells
Time [h]
F.
Control
Palbociclib
Fraction of G1 + G1S cells
Time [h]
G.
G1
S
G2
Control
Palbociclib
Density
DAPI
Geminin Fluorescence
Cdt1 Fluorescence
H.
Control
Palbociclib
48h
72h
96h
120h
Phase
G1
G1S
S
G2M
Fraction
I.
DAPI
EdU
Control
Palbociclib
J.
DAPI
Caspase
Control
Palbociclib
K.
G1
S
G2
Control EdU
Palbociclib EdU
Density
DAPI
no EdU
Control EdU
Palbociclib EdU
Count
EdU Fluorescence
L.
EdU+ proportion
0.06%
64.03%
29.96%
no EdU
Control EdU
Palbociclib EdU
Conditions
M.
EdU Fluorescence
Control EdU
Palbociclib EdU
Conditions
N.
Caspase/DAPI ratio
Control
Palbociclib
Conditions
O.
Ccnd2
Ccnd1
D-Cyclins
Ccne1
Trh
Npm1
Igfbp5
−log10(p)
log2 Fold Change
Cell Cycle G1/S Phase Transition
Mitotic Cell Cycle Phase Transition
Regulation Of Cyclin-Dependent Protein Kinase Activity
G1/S Transition Of Mitotic Cell Cycle
−log10(p)
P.
Control
Palbociclib
Normalized log CPM
***
**
***
**
*
*
Ccnd1
Ccnd2
Pcna
Ccna2
Mki67
Mcm6

Figure S2. Cell-cycle characterisation of Palbociclib-treated gastruloids.

(A) Relative cell number in 2D mESCs cultured in 2i/LIF medium under control and Palbociclib-treated conditions, showing a dose-dependent reduction in cell proliferation following treatment. Mean $\pm$ SD.
(B) Representative images of pRB staining in mESC colonies cultured in 2i/LIF medium under control and 5 μM Palbociclib-treated conditions.
(C) Quantification of colony size and pRB staining in mESCs cultured in 2i/LIF medium under control and 5 μM Palbociclib-treated conditions, showing reduced colony size and pRB levels following treatment. Significance from a two-sided Welch's t-test on log10-transformed data: $p < 0.05$ *, $p < 0.01$ **, $p < 0.001$ ***, $p < 0.0001$ ****.
(D)Fucci(CA) imaging of gastruloid across time allows characterization of cell-cycle phase distributions in Control and CDK4/6 inh. gastruloid. Red cells are in the G1 phase, green cells in the S phase and orange cells are in the G2M phase.
(E) Quantification of cell number from Fucci(CA) gastruloid images shows a growth arrest under 5 μM CDK4/6 inhibition.
(F) Quantification of cell cycle state from Fucci(CA) gastruloid images shows a significant, time-dependent increase in G1/G1–S cell proportions under 5 μM CDK4/6 inhibition.
(G) Flow cytometry fluorescence histograms (DAPI, GEMININ, CDT1) allows characterization of the population cell-cycle state in control and Palbociclib-treated gastruloid; black dotted lines indicate positivity thresholds.
(H) Cell cycle phase proportions quantified from flow cytometry confirm enrichment of G1 and G1/S cells and reduction of S and G2/M cells in the cell cycle inhibited condition.
(I) Representative EdU incorporation imaging in 120h gastruloid reveals reduced incorporation under CDK4/6 inhibition compared with control.
(J) Representative Caspase stainings illustrating the identification of apoptotic cells in control and CDK4/6 inh. gastruloid.
(K) Flow cytometry fluorescence histograms (DAPI, EdU) identify EdU-positive cell population; black dotted lines indicate positivity thresholds.
(L) Quantification of EdU-positive fractions shows a significant decrease under CDK4/6 inhibition compared to the control condition.
(M) Distribution of EdU fluorescence in EdU-positive cells shows a shift to lower intensities under CDK4/6 inhibition compared with controls, consistent with slower S-phase progression.
(N) Caspase stainings quantification shows a higher, but variable, fraction of dying cells under inhibition. n=4 in each condition.
(O) Differential expression and GO term analysis of bulk RNA-seq from individual gastruloids reveal differences in cell-cycle pathways between conditions.
(P) Normalised log-CPM of cell-cycle genes shows up-regulation of D-cyclins (G1) and down-regulation of proliferative markers (e.g., *Mki67*, *Pcna*) with cell cycle inhibition. $n_{control}$ =7, n_{others} = 4. Significance from a two-sided Welch's t-test on normalized log CPM: $p < 0.05$ *, $p < 0.01$ **, $p < 0.001$ ***, $p < 0.0001$ ****.
All box plots show median, 25th/75th percentiles (box), and 1.5$\times$ IQR whiskers.

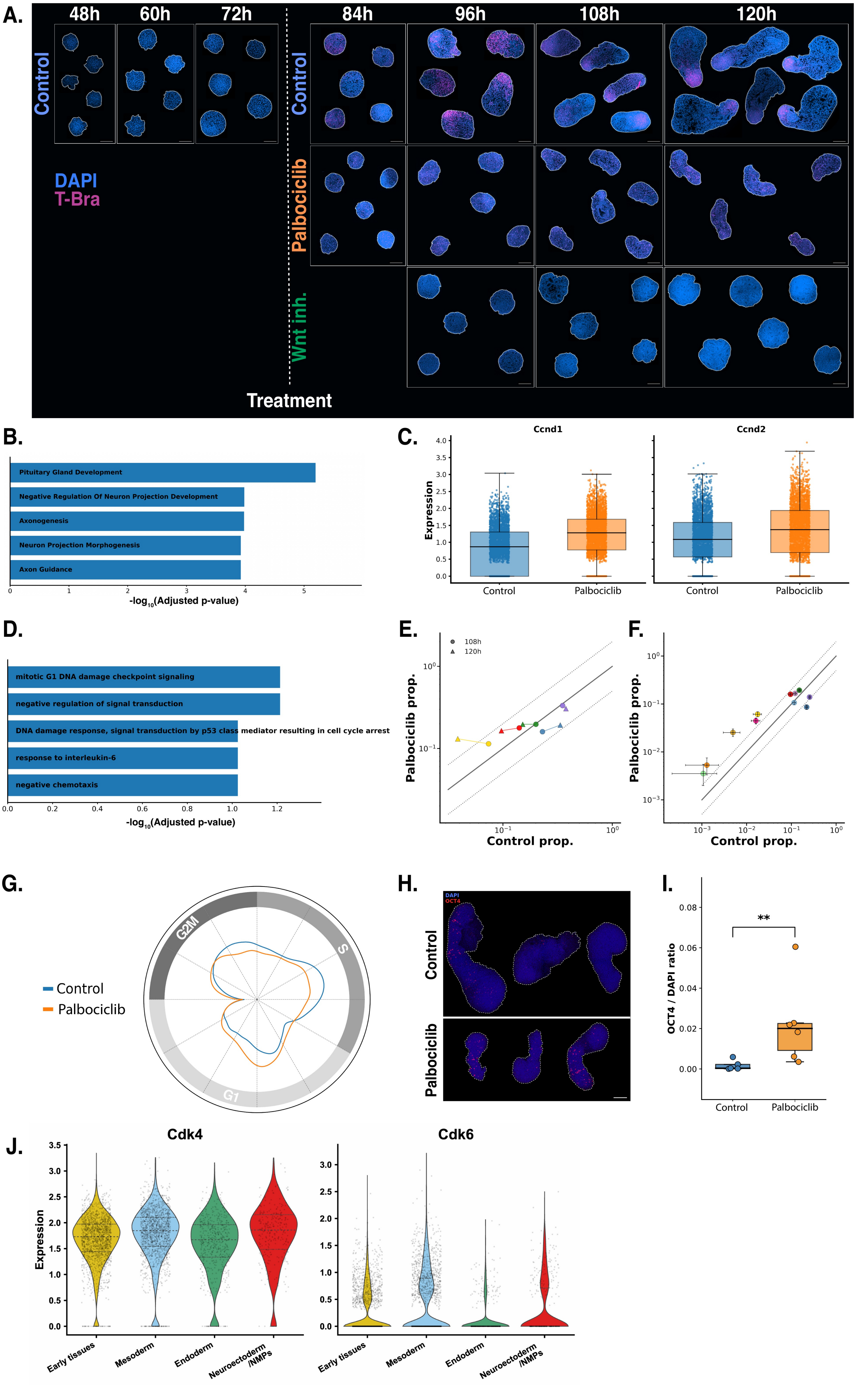
A.
48h
60h
72h
84h
96h
108h
120h
Control
Control
Palbociclib
Wnt inh.
DAPI
T-Bra
Treatment
B.
Pituitary Gland Development
Negative Regulation Of Neuron Projection Development
Axonogenesis
Neuron Projection Morphogenesis
Axon Guidance
-log10(Adjusted p-value)
C.
Ccnd1
Ccnd2
Expression
Control
Palbociclib
Control
Palbociclib
D.
mitotic G1 DNA damage checkpoint signaling
negative regulation of signal transduction
DNA damage response, signal transduction by p53 class mediator resulting in cell cycle arrest
response to interleukin-6
negative chemotaxis
-log10(Adjusted p-value)
E.
108h
120h
Palbociclib prop.
Control prop.
F.
Palbociclib prop.
Control prop.
G.
Control
Palbociclib
G2M
S
G1
H.
DAPI
OCT4
Control
Palbociclib
I.
**
OCT4 / DAPI ratio
Control
Palbociclib
J.
Cdk4
Cdk6
Expression
Early tissues
Mesoderm
Endoderm
Neuroectoderm /NMPs
Early tissues
Mesoderm
Endoderm
Neuroectoderm /NMPs

Figure S3. Characterisation of single-cell transcriptomic responses to CDK4/6 and Wnt inhibition in developing gastruloids.

(A) Representative T-mCherry–expressing gastruloids from the same batches used for scRNA-seq, shown at the time of collection.
(B) Enrichment of neuronal GO terms in the PORCN-inhibited cluster indicates Wnt inhibition promotes neuronal differentiation.
(C) D-Cyclin overexpression under CDK4/6 inhibition confirmed by scRNA-seq.
(D) GO term analysis of CDK4/6 inhibitors–upregulated genes validates G1 cell-cycle deregulation.
(E) Comparison of cell-type proportions between control and CDK4/6 inhibitor conditions at 108 h and 120 h, shown on a logarithmic scale, revealing a rebalancing of cell populations that increases with time of treatment. Mean $\pm$ SD.
(F) Comparison of cell-type proportions between control and CDK4/6 inhibitor conditions at 120 h, with error bars calculated by bootstrapping cells within the sample.
(G) θ-space phase density confirms an excess of G1-phase cells under CDK4/6 inhibition.
(H) Representative Oct4 staining in 120 h gastruloids under control and Palbociclib-treated conditions.
(I) Quantification of Oct4 staining in 120 h gastruloids under control and Palbociclib-treated conditions, showing an increased proportion of Oct4-positive cells following treatment. Significance from a two-sided Welch's t-test: $p < 0.05$ *, $p < 0.01$ **, $p < 0.001$ ***, $p < 0.0001$ ****.
(J) Log-transformed Cdk4 and Cdk6 expression across cell lineages, showing broadly uniform expression across the identified populations.
All box plots show median, 25th/75th percentiles (box), and 1.5$\times$ IQR whiskers. The violin plot shows the density estimate; lines mark min/max.

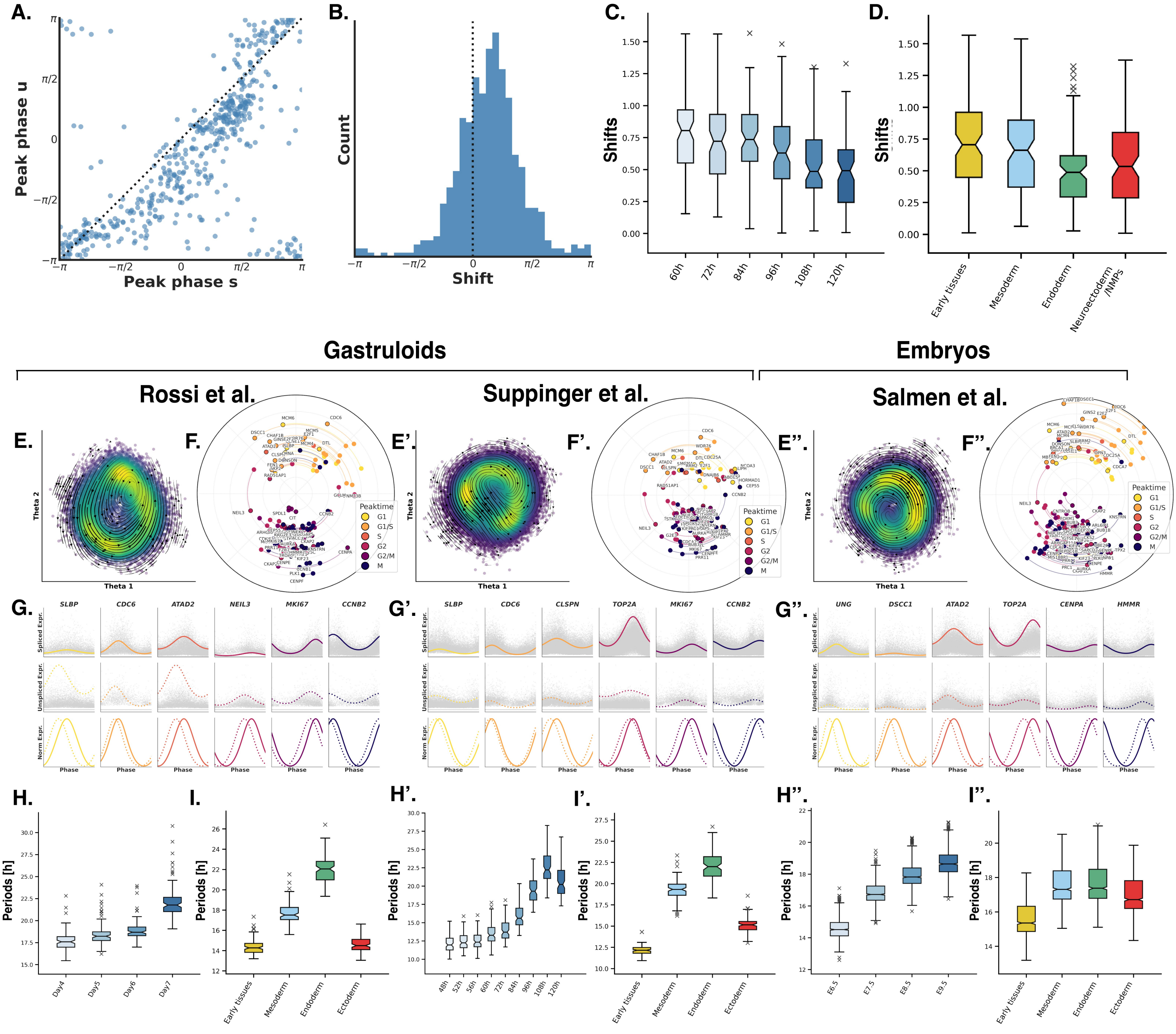

Figure S4. Characterisation and validation of cell cycle period mapping across datasets.

(A) Scatter plot of unspliced versus spliced RNA peak times, with most genes falling on one side of the diagonal, indicating consistent phase shifts between the two.
(B) Distribution of phase shifts, with most genes exhibiting shifts between 0 and $\pi/2$.
(C) Phase shift distribution over time reveals a negative correlation between phase shift and differentiation time, consistent with cell cycle lengthening during differentiation.
(D) Phase shift distribution across cell types highlights distinct patterns among cell types.

For the Rossi et al.[S1], Suppinger *et al.*[S2] ('), and Salmen et al.[S3] ('') datasets:

(E) Projection of cells in the inferred circular θ-space obtained from the variational autoencoder model and RNA velocities trajectories.
(F) Polar plots displaying unspliced and spliced (outlined) transcript profiles by peak phase (angle) and amplitude (radius); arcs link corresponding spliced and unspliced counts of the same gene, and colours indicate annotated cell cycle phases.
(G) Unspliced and spliced RNA expression profiles of cyclic genes plotted as a function of cell cycle phase.
(H, I) Boxplots and scatter plots showing inferred cell cycle period distributions and corresponding cell velocities across cell types and lineages.
All box plots show median, 25th/75th percentiles (box), and 1.5$\times$ IQR whiskers.

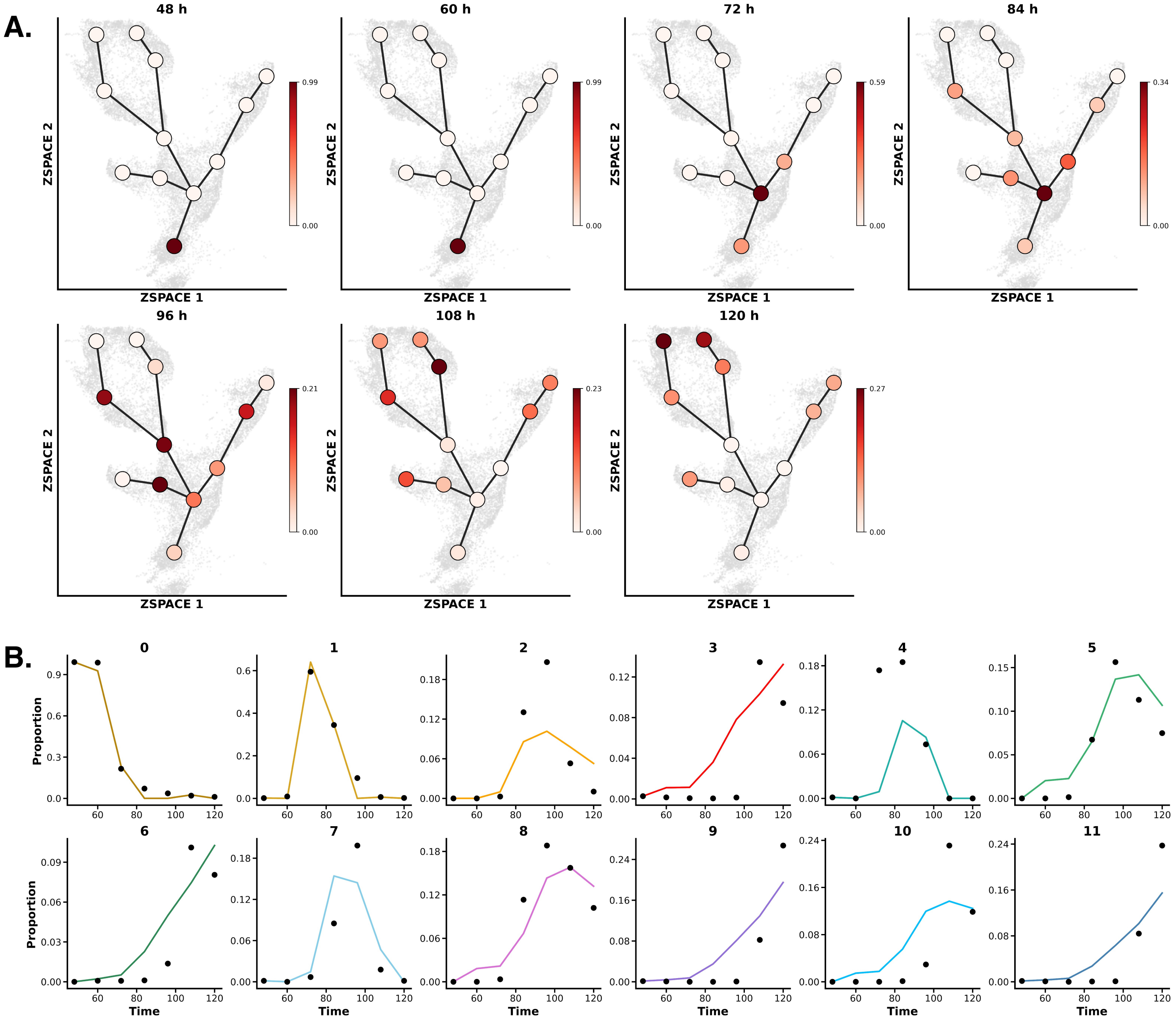

Figure S5. Simulation of Population Dynamics Under Control and Growth Arrest Conditions.

(A) Temporal evolution of observed population densities across UMAP-defined node compartments in the Control condition. (B) Observed control proportions are shown as black dots, with their simulated fits plotted as coloured lines over time. Numbers correspond to nodes illustrated in **Figure 5B**.

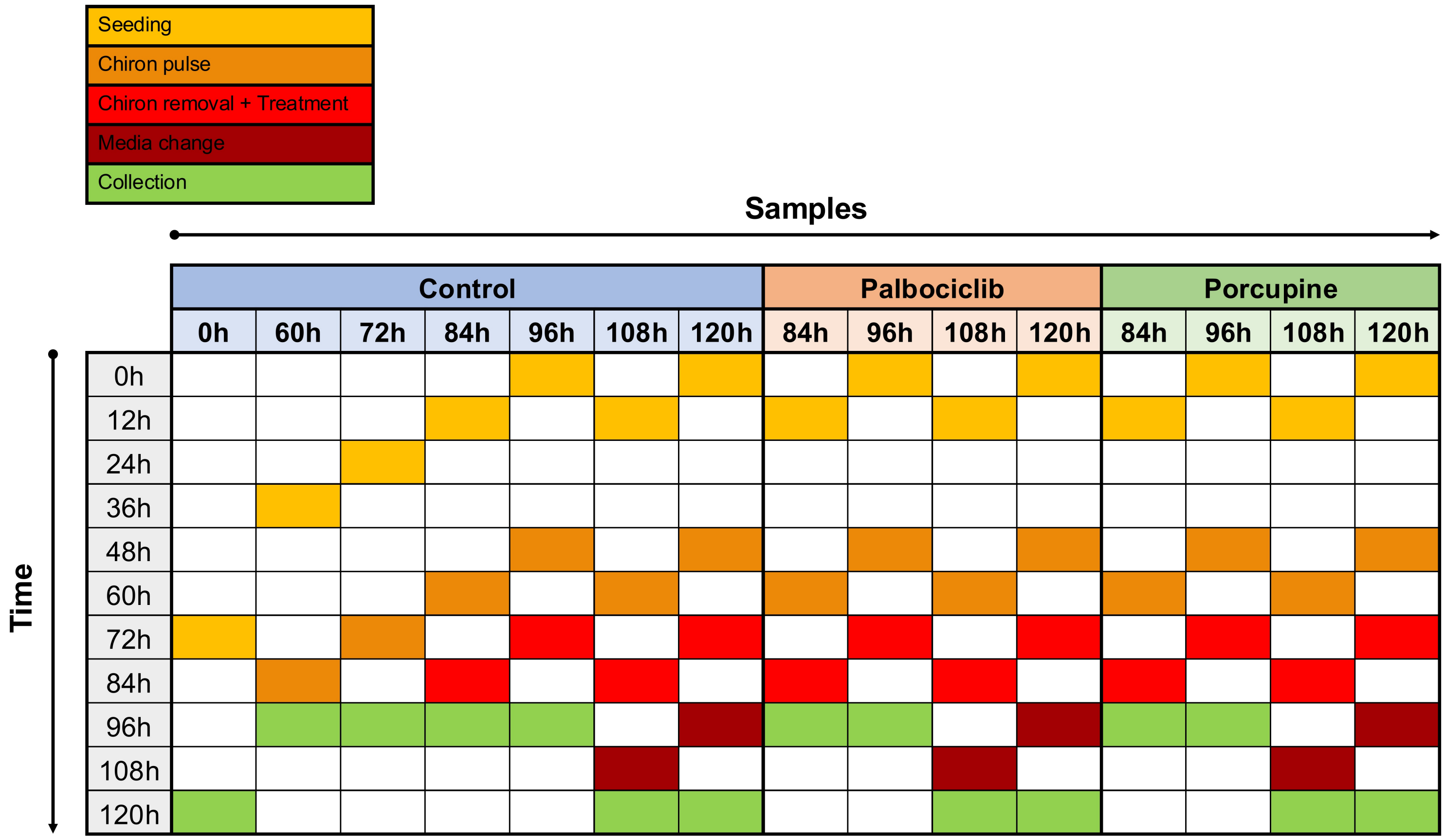

Figure S6. Sampling and treatment schedule of the scRNA-seq time course, related to STAR Methods.
Timing of seeding, Chiron pulse, Chiron removal and treatment, media changes and collection for each sample of the Control, Palbociclib and Porcupine inhibitor conditions.

Category	Gene(s)
CDKs/Cyclins	*Ccnd1*, *Ccnd2*, *Ccne1*, *Ccnt1*, *Cdkn1a* (p21)
DNA replication	*Mcm6*, *Pola2*, *Prim2*, *Lig1*, *Pcna*, *Dtymk*
Mitosis	*Aurkc*, *Ncapd2*, *Aspm*, *Anapc11*, *Fam111a*, *Rec8*
Checkpoint response	*Chaf1a*, *Gadd45gip1*, *Btg2*
Transcription factors	*Foxm1*, *Mycn*, *Klf4*, *Klf5*, *Klf9*, *Hey1*, *Tfcp2l1*, *Tfap2c*
Stress / apoptosis	*Ccn2*, *Pdgfa*, *Birc6*, *Sat1*/*Sat2*, *Hspb1*, *Gpx1*, *Gpx4*

Table S1. **Differentially expressed genes from bulk RNA-seq comparing control and Palbociclib-treated conditions indicate disruption of cell-cycle regulation.**

Category	Gene(s)
Cell cycle regulators	*Ccnd1*, *Ccnd2*, *Cdk8*, *Ccng1*, *Gtse1*, *Plk2*, *Zw10*, *Cdkl3*
p53 signalling	*Rb1*, *Mdm2*, *Trp53inp1*, *Trp53i11*, *Trp53cor1*, *Phlda3*, *Zmat3*, *Pidd1*, *Ccng1*, *Ei24*, *Btg2*, *Ddit4*, *Ddit4l*
DNA damage response & repair	*Polk*, *Mgmt*, *Capn2*, *Atrn*, *Sesn3*, *Ddias*, *Hdac9*, *Fbxl20*
Signalling pathways	*Notch1*, *Ptch1*, *Stat3*, *Tgfbr3*, *Nr3c1*, *Socs2*, *Csnk1g1*, *Map3k20*, *Pik3r3*
Apoptosis	*Apaf1*, *Tnfrsf10b*, *Perp*, *Emp3*, *Sat1*, *Sesn3*, *Tnfrsf19*
Cell division	*Cep170b*, *Kif1c*, *Sept8*, *Zw10*, *Nup93*, *Capn2*

Table S2. **Genes driving classification of control vs Palbociclib-treated cells in scRNA-seq data reflect cell-cycle and p53-related responses.** Classification was performed using logistic regression for each cell type, and the list represents the most recurrent genes across lineages.

Category	Gene(s)
Neural fate specification	*Sox2*, *Sox3*, *Otx2*, *Pou3f1*, *Pou5f1*, *Tfap2a*, *Hesx1*, *En1*, *Lmx1b*, *Six3*, *Pax2*, *Zic5*, *Sall2*, *Bcl11a*
Axon connectivity	*Ephb1*, *Dcc*, *Nrcam*, *Nfasc*, *Celsr2*, *Cdh10*, *Cdh20*, *Plxnc1*, *Astn1*, *Adgrv1*, *Prune2*
Neurite outgrowth	*Stmn2*, *Stmn3*, *Crmp1*, *Ina*, *Tubb3*, *Tubb4a*, *Mapk8ip2*, *Camkv*, *Dp10*, *Sez6*
Synapse signalling	*Snap25*, *Grin2b*, *Nrxn3*, *Sorcs1*, *Lpar4*, *Gngt2*, *Dgkb*, *Plce1*, *Cacng4*, *Cacng7*, *Kcnj3*

Table S3. **Wnt inhibition leads to an increased neuronal signature in 120 h gastruloids compared to controls.**

Cell type	Fold change	log2(FC)	p-value	p-value (Bonferroni)
Naive pluripotency	—	—	1.00e+00	1.00e+00
**Epiblast**	**5.14**	**2.36**	**1.00e-03**	**1.20e-02**
**Primitive streak**	**3.47**	**1.80**	**1.00e-03**	**1.20e-02**
Early cardiac mesoderm	0.92	-0.11	1.78e-01	1.00e+00
**Cardiac mesoderm**	**0.39**	**-1.35**	**1.00e-03**	**1.20e-02**
**Early somitic mesoderm**	**1.37**	**0.45**	**1.00e-03**	**1.20e-02**
**Somitic mesoderm**	**0.55**	**-0.87**	**1.00e-03**	**1.20e-02**
Early endoderm	3.29	1.72	6.00e-03	7.20e-02
**Endoderm**	**1.29**	**0.36**	**1.00e-03**	**1.20e-02**
**Early neuroectoderm/NMPs**	**4.11**	**2.04**	**1.00e-03**	**1.20e-02**
**Neuroectoderm/NMPs**	**1.67**	**0.74**	**1.00e-03**	**1.20e-02**
**Wnt inh. state**	**2.77**	**1.47**	**1.00e-03**	**1.20e-02**

Table S4. **Bootstrap resampling of cell-type proportions confirms robust shifts in most lineages following CDK4/6 inhibition.** Fold change and log2(fold change) in cell-type proportion (Palbociclib-treated vs. control, 120 h) were computed with the proportion averaged across 2,000 bootstrap resamples of the scRNA-seq dataset; p-values were obtained from the bootstrap distribution and corrected for multiple comparisons using the Bonferroni method. Bold rows indicate lineages with a significant shift after correction ($p < 0.05$).

Supplemental references

[S1] Rossi, G., Broguiere, N., Miyamoto, M., Boni, A., Guiet, R., Girgin, M., Kelly, R.G., Kwon, C., and Lutolf, M.P. (2021). Capturing Cardiogenesis in Gastruloids. Cell Stem Cell *28*, 230–240.e6. URL: https://linkinghub.elsevier.com/retrieve/pii/S1934590920305075. doi: 10.1016/j.stem.2020.10.013.

[S2] Suppinger, S., Zinner, M., Aizarani, N., Lukonin, I., Ortiz, R., Azzi, C., Stadler, M.B., Vianello, S., Palla, G., Kohler, H., Mayran, A., Lutolf, M.P., and Liberali, P. (2023). Multimodal characterization of murine gastruloid development. Cell Stem Cell *30*, 867–884.e11. URL: https://linkinghub.elsevier.com/retrieve/pii/S1934590923001704. doi: 10.1016/j.stem.2023.04.018.

[S3] Salmen, F., De Jonghe, J., Kaminski, T.S., Alemany, A., Parada, G.E., Verity-Legg, J., Yanagida, A., Kohler, T.N., Battich, N., Van Den Brekel, F., Ellermann, A.L., Arias, A.M., Nichols, J., Hemberg, M., Hollfelder, F., and Van Oudenaarden, A. (2022). High-throughput total RNA sequencing in single cells using VASA-seq. Nature Biotechnology *40*, 1780–1793. URL: https://www.nature.com/articles/s41587-022-01361-8. doi: 10.1038/s41587-022-01361-8.
